# Effect of early-life protein supplementation on childhood obesity and related metabolic outcomes: a systematic review and meta-analysis

**DOI:** 10.1016/j.ajcnut.2025.09.017

**Published:** 2025-09-13

**Authors:** Rukman Manapurath, Christopher P Duggan, Ranadip Chowdhury, Sunita Taneja, Nita Bhandari, Tor A Strand

**Affiliations:** 1 Centre for International Health, Department of Global Public Health and Primary Care, University of Bergen, Bergen, Norway; 2 Department of Maternal and Child Health - Nutrition, Society for Applied Studies, New Delhi, India; 3 Division of Gastroenterology, Hepatology, and Nutrition, Boston Children's Hospital, Boston, MA, United States; 4 Departments of Nutrition and Global Health and Population, Harvard T.H. Chan School of Public Health, Boston, MA, United States; 5 Department of Research, Innlandet Hospital Trust, Lillehammer, Norway

**Keywords:** protein supplementation, childhood obesity, body composition, complementary feeding, meta-analysis, growth outcomes, insulin-like growth factor, randomized controlled trial, low- and middle-income countries, systematic review

## Abstract

**Background:**

Protein intake during complementary feeding plays a vital role in childhood growth. However, the effects of varying protein quantity and source on obesity and related metabolic outcomes remain uncertain.

**Objectives:**

This study aimed to evaluate the effect of protein intake (quantity and source) on childhood obesity [weight-for-length *Z*-score (WLZ) > 2], body composition [fat mass (FM), fat-free mass (FFM)], and metabolic markers (insulin, insulin-like growth factor-1) in children aged 6–23 mo, including outcomes beyond 24 mo where available.

**Methods:**

A systematic search was conducted in MEDLINE, Cochrane CENTRAL, Embase, Web of Science, and CINAHL (January 2000–May 2024) for randomized controlled trials that compared high with low protein intake (diets with higher protein defined as an absolute difference of ≥5 g/d between groups), animal compared with plant-based protein, and meat compared with dairy. Random-effects meta-analyses were performed, and heterogeneity was assessed using *I*^2^ statistics. Owing to the lack of binary obesity outcome data, WLZ was analyzed as a continuous measure to assess shifts in weight-for-length distribution.

**Results:**

Of 5817 records identified, 20 publications (from 19 trials) met the inclusion criteria, with 12 included in the meta-analysis. Diets with higher protein content did not substantially affect WLZ. Standardized mean difference (SMD) was 0.0 (95% CI: –0.09, 0.09) for low- and middle-income countries and 0.17 (95% CI: –0.12, 0.47) for high-income countries. No effects were found for FFM [SMD: –0.05 (95% CI: –0.31, 0.21)] or FM [SMD: 0.17 (95% CI: –0.41, 0.74)]. We were not able to identify any effects of protein source of any of the outcomes. Insufficient data precluded meta-analysis for metabolic markers.

**Conclusions:**

There is limited evidence of any impact of protein quantity or quality during complementary feeding on growth or body composition. Given the short-lasting exposures and follow-up times, the long-term metabolic effects of protein supplementation require further investigation.

This trial was registered at PROSPERO as CRD42024550409.

## Introduction

Protein is an essential macronutrient, playing a crucial role in child growth and development [1]. The complementary feeding period, from 6 to 23 mo of age, represents a critical developmental window during which infants and young children transition from exclusive human milk feeding to a diversified diet that incorporates family foods alongside human milk. Emerging evidence suggests that protein intake during this period influences growth patterns, including linear growth and weight gain [2]. Although chronic inadequate intake can contribute to undernutrition and stunting, excess protein intake, particularly during infancy, has been associated with an increased risk of overweight and obesity in early childhood [[3], [4], [5], [6]]. However, much of the existing evidence linking higher protein intake to obesity risk comes from studies in formula-fed infants, particularly in high-income settings. Given that complementary feeding occurs alongside both human milk and formula feeding in diverse settings, it is important to evaluate whether similar associations hold across different feeding patterns and contexts.

Protein intake may also influence metabolic pathways beyond its role in growth. Animal-based proteins, particularly those rich in branched-chain amino acids, stimulate insulin and insulin-like growth factor-1 (IGF-1) production, which are involved in nutrient utilization and muscle growth [7]. Although these pathways support linear growth, some studies suggest that higher protein intake, especially from dairy, may also be associated with increased adiposity risk [2,8]. However, findings remain inconsistent, with other studies reporting no significant differences in adiposity based on protein intake or source [9].

Global nutrition guidelines, including those from the FAO/WHO and the Dietary Reference Intakes from the Institute of Medicine, provide recommendations for protein intake in infants and young children [10,11]. These guidelines suggest a protein intake of 1.12–0.7 g/kg/d for children aged 6–23 mo, depending on growth and energy requirements. However, there is an ongoing debate over whether low protein during complementary feeding intake raises risk of undernutrition, whereas excess, especially from animal sources, may increase obesity risk. This systematic review and meta-analysis aimed to evaluate the effect of protein intake (both quantity and source) on childhood obesity, body composition, and metabolic markers in children aged 6–23 mo.

## Methods

This systematic review and meta-analysis was conducted according to the standard methods for systematic reviews of intervention studies. Reporting of the review follows the PRISMA guidelines. The review protocol was registered with the PROSPERO (CRD42024550409).

### Outcomes

The primary outcome was the proportion of children with overweight or obesity, defined as weight-for-length/height *Z*-scores (WLZ) > 2 based on WHO growth standards [12]. Owing to the limited direct reporting of children with overweight or obesity in the included trials, we used WLZ as a proxy to evaluate growth patterns and adiposity. Secondary outcomes included, when available, body composition indicators such as fat mass (FM) and fat-free mass (FFM). For this review, metabolic biomarkers refer specifically to measurable blood concentrations including insulin, IGF-1, and IGF-binding protein-3 (IGFBP-3) that are pivotal in regulating growth, glucose metabolism, and adiposity.

### Eligibility criteria

Studies were eligible for inclusion if they were randomized controlled trials (RCTs) evaluating the effects of protein quantity or source (animal-based or plant-based) during the complementary feeding period (intervention) compared with lower protein intake, alternative protein sources, or standard diets (comparator). Only RCTs were eligible (study design). Trials were primarily required to include infants and young children aged 6–23 mo (population); however, studies that initiated interventions slightly earlier (from 4 mo of age) were also included if the intervention was intended to support or transition into complementary feeding. To be included, studies had to report outcomes on WLZ, BMI-for-age Z-score, overweight/obesity incidence, body composition measures such as FM or FFM, or metabolic biomarkers—insulin or IGF-1/IGFBP3 (outcomes). Additionally, studies were required to provide sufficient data for calculating estimates, such as means and SD for relevant groups. Studies were excluded if they did not report ≥1 of the predefined outcomes or if they focused on the treatment of acute or severe malnutrition or involving children with chronic illnesses (e.g., chronic kidney disease) or if only 1 arm received disproportionately high fat content (defined as a fat intake exceeding 40% of total energy intake, provided exclusively to 1 intervention arm without similar concentrations in the control or comparison groups).

### Literature search

A comprehensive search of literature was conducted through MEDLINE, Embase, Web of Science, the Cochrane Central Register of Controlled Trials, and CINAHL(EBSCO) for studies published between 1 January, 2000, and 31 May, 2024. The search strategy included terms such as “protein intake,” “animal protein,” “plant protein,” “weight-for-height-Z-score,” “BMI-for-age Z-score,” “overweight,” “obesity,” and “body composition.” The reference lists of relevant articles and reviews were manually searched to identify additional eligible studies. The detailed search strategy is provided in Supplemental Table 1.

### Study selection and data extraction

Search results from each database were imported into Covidence reference management software [13], and duplicates were removed. Title and abstract screening, as well as full-text review, were conducted independently and in duplicate by 2 reviewers (RM, RC). Any disagreements at either stage were resolved through discussion or, if needed, by consulting the 2 senior reviewers (TS, CD).

Data from the included studies were extracted using a standardized data extraction form in Microsoft Excel by 2 independent reviewers (RM, RC). Extracted data included study characteristics such as study ID, country, funding information, and population; details of intervention and control groups, including protein quantity and source; age groups and duration of intervention; and outcome measures, such as anthropometric measures, body composition, and biomarkers. This review prioritized prospective trials that specifically addressed protein supplementation, as observational studies could not reliably isolate the effects of standardized interventions from natural dietary variations. For studies with missing or unclear data, we attempted to contact the corresponding authors by e-mail to obtain additional information.

Exposures of interest in this review were defined according to the key intervention contrasts reported in the included studies: *1*) high compared with low total protein intake or supplementation; *2*) meat-based compared with dairy-based protein sources; and *3*) animal-based compared with plant-based protein sources. Studies were categorized as high or low protein supplementation based on the absolute difference in protein intake between intervention groups. Given the variation in how protein intake was reported across studies—either as total daily intake (dietary modification studies) or additional supplementation (supplement-based interventions)—we used an operational definition of high-protein intake as an absolute difference of ≥5 g/d between groups. The 5 g/d threshold corresponds to ∼38%–45% of the daily protein requirement for children aged 6–23 mo, based on WHO/FAO estimates. Studies in which additional macronutrient components (such as fat or energy content) were balanced across intervention and control groups were retained. Although the primary exposure period was during complementary feeding (6–23 mo), there was no restriction on the duration of follow-up after the intervention.

### Data synthesis and statistical analysis

Studies were grouped for analysis based on protein quantity (e.g., higher concentrations and lower concentrations) and protein source (animal-based and plant-based). Where necessary, overall protein intake was recalculated using dietary intake if sufficient data were available, to ensure that comparisons reflected the actual protein energy contributions.

WLZ was analyzed as a continuous variable to reflect overall growth patterns. For studies with >2 intervention groups, those with comparable differences in protein quantity or quality were selected for meta-analysis to maintain consistency across comparisons. For studies in which the protein energy ratio (PE%) was not explicitly reported, we calculated it manually using dietary recall data and the composition of nutrients provided in the intervention and control diets.

Meta-analyses were conducted using random-effects models to account for variability among studies [14]. As direct dichotomous outcomes (e.g., WLZ > +2 SD) were not consistently reported, proportions were estimated from continuous data using statistical formulas based on the Z distribution. Pooled standardized mean differences (SMDs) and 95% confidence intervals (CIs) were calculated for each group, with SMD used to compare effects. Heterogeneity was assessed using the *I*^2^ statistic, with values >50% indicating substantial heterogeneity [15]. For studies that reported effect estimates and 95% CIs, SMDs were calculated to ensure consistency across studies. For studies reporting mean differences and corresponding 95% CIs between intervention and control groups, SMDs were calculated directly using the mean difference (Δ$\bar{X}$) and pooled SD (SD_p_). The pooled SD (SD_p_) was derived from the SDs of the intervention and control groups. Meta-analyses were performed using Stata (version 18, StataCorp), employing the “meta” command for continuous outcomes. Meta-analyses were performed only when 2 or more studies reported the outcome of interest.

Subgroup analyses were conducted to investigate study-level characteristics, including the income classification of the countries (low-to-middle-income or high-income) based on the World Bank income groups at the time of the study. Studies evaluating high- compared with low-protein intake were classified into 2 subgroups: dietary modification studies, in which total protein intake differed because of changes in habitual diet, and supplement-based interventions, in which only the additional protein from supplementation was considered, whereas baseline diets were similar between groups. These subgroups were analyzed separately in the meta-analysis.

### Risk of bias assessment

The risk of bias for included studies was assessed using the Cochrane Risk of Bias tool for RCTs, tailored for both individually randomized and cluster-randomized designs [16]. This tool evaluates potential biases across several domains, including the randomization process, deviations from intended interventions, missing outcome data, measurement of outcomes, and selection of reported results. Each study was independently assessed by 2 reviewers (RM and RC), with disagreements resolved by 2 senior reviewers (CPD and TAS). Studies were classified as having low, high, or some concerns regarding risk of bias, with results incorporated into the overall interpretation of findings.

### Quality assessment

The quality of evidence for each outcome was assessed using the Grading of Recommendations, Assessment, Development, and Evaluation (GRADE) approach [17]. This framework evaluates evidence across 5 domains: risk of bias, inconsistency, indirectness, imprecision, and publication bias. The evidence quality was downgraded where substantial bias, heterogeneity, or imprecision was identified.

## Results

### Study selection

**FIGURE 1:**
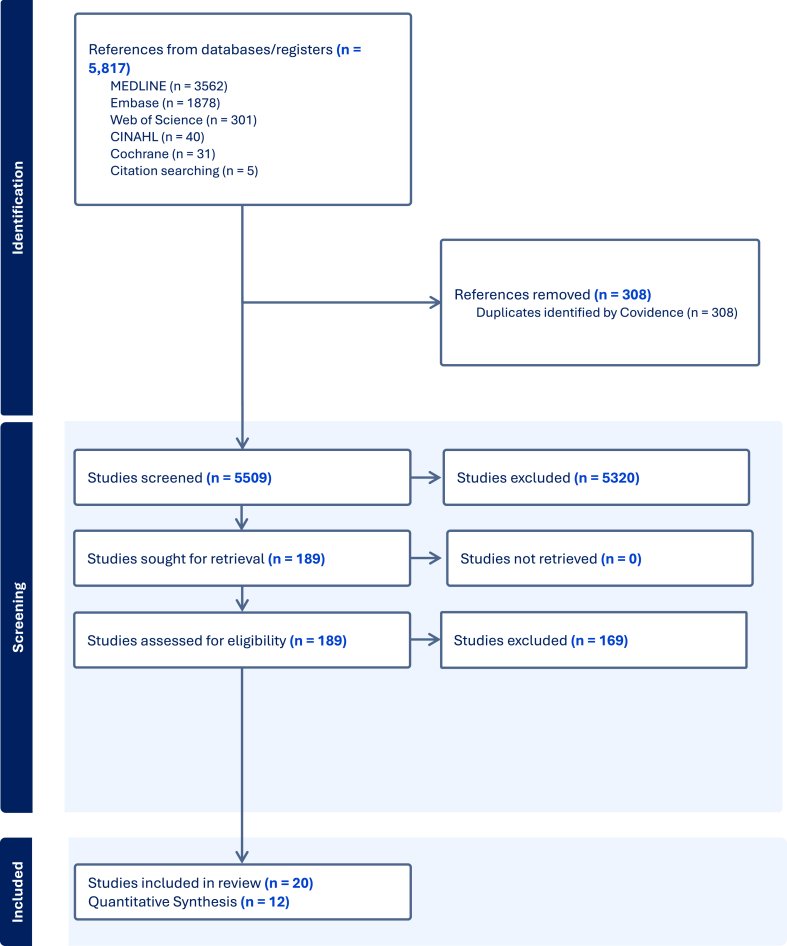
This figure presents the study selection process following PRISMA guidelines, including the number of records identified, screened, excluded, and included in the final systematic review and meta-analysis. In all forest plots, sample sizes for intervention and control groups are shown in parentheses. Summary estimates are displayed only for groups with 2 or more studies. The outcome is expressed as the standardized mean difference.

A total of 5817 records were identified through database searches, including MEDLINE (*n =* 3562), Embase (*n =* 1878), Web of Science (*n =* 301), CINAHL (*n =* 40), and the Cochrane Central Register of Controlled Trials (*n =* 31). An additional 5 records were retrieved through citation searching. After duplicate removal (*n =* 308), 5509 records underwent title and abstract screening, from which 189 studies were selected for full-text review. Of these, 169 were excluded for reasons detailed in the Supplementary data. The main reasons included observational studies (*n =* 29), review articles (*n =* 22), cohort studies (*n =* 18), interventions starting at 2 mo (*n =* 14) or after birth (*n =* 6), enrollment of children under 6 mo (*n =* 13), ongoing studies or protocols (*n =* 6), commentary/editorials (*n =* 5), lack of appropriate comparison group (e.g., lipid-based nutrient supplements' (LNS) LNS with fortified micronutrient supplement, *n =* 7), LNS supplement trials with disproportionately high fat (>40%) in 1 group (*n =* 3), studies focused on acute malnutrition (*n =* 3), multidomain interventions (*n =* 3), studies conducted before 2000 (*n =* 3; reflecting the period after international guidelines shifted from recommending exclusive human milk feeding for 4 mo to the current 6-mo recommendation, and to include contemporary complementary feeding practices and interventions), and a range of other specific reasons (e.g., studies in children with chronic illness, insufficient data, duplicate reports, or no response from authors after contact). A full list of excluded studies and reasons is provided in Supplemental Table 2. Twenty studies met all inclusion criteria and were included in the systematic review, with 12 provided sufficient quantitative data for meta-analysis. The remaining 8 studies were included in the narrative synthesis only because they either did not report the required outcome measures, reported outcomes in an incompatible format (graphs), or lacked the necessary statistical data (e.g., means, SDs, or effect estimates) for inclusion in the pooled meta-analyses. The PRISMA flow diagram (Figure 1) summarizes the study selection process.

### Study characteristics

The included studies evaluated interventions in infants and young children primarily aged 6–23 mo and assessed the effects of varying protein quantities and sources on WLZ, overweight/obesity incidence, body composition, and metabolic biomarkers. Of the 20 studies, 12 evaluated the effects of varying protein quantities, comparing high-protein to low-protein diets [[18], [19], [20], [21], [22], [23], [24], [25], [26], [27], [28], [29]]. Five studies compared meat-based protein to dairy-based protein in complementary feeding interventions [24,[30], [31], [32], [33]]. Five studies investigated the differential effects of animal-based protein compared with plant-based protein [9,25,[34], [35], [36]]. Study populations spanned low- and middle-income countries (LMICs) as well as high-income settings, with interventions ranging from fortified complementary foods to specific protein-rich dietary supplements. Study characteristics, including intervention details and population demographics, are summarized in Table 1 [9,[18], [19], [20], [21], [22], [23], [24], [25], [26], [27], [28], [29], [30], [31], [32], [33], [34], [35], [36]]. Refer to Supplemental Material section 2 for detailed results of the studies included. For high and low protein intake comparison, to ensure comparability, both absolute protein intake (g/day) and PE%, whenever available (g/100 kcal), were reported. See Supplemental Table 3 for details.

**TABLE 1:** Details of included studies.

Study details	Inclusion criteria	Age group (mo)	Baseline, sample size, follow-up rate	Comparison type	Group 1	Group 2	Duration of intervention (mo)	Outcomes
Johansson et al. [21], 2023, Sweden, parallel group, open-label	Healthy, singleton 4- to 6-mo-old infants, exclusively human milk fed and/or formula fed at the time of recruitment, born after >37 wk of gestation and with birth weight >2500 g	4–6	Normally nourished, 250, 82%	High vs. low protein	Conventional group: Swedish dietary recommendations with overall 28.4 g/d	Nordic homemade baby food recipes, protein-reduced baby food products with overall 20 g/d (reduced protein)	6	WLZ, FM, FFM (deuterium dilution)
Taneja et al. [28], 2022, India, parallel group	Infants aged 6 mo, human milk fed, not severely malnourished (WLZ < −3 SD)	6	20% Stunting, 19% underweight, 1548, >95%	High vs. low protein	Isocaloric milk cereal mix supplement (5.6 g/125 kcal) fortified with 1 RDA essential micronutrients	No supplementation	6	WLZ, FM, FFM (deuterium dilution)
Konyole et al. [22], 2019, Kenya, parallel group, double-blind	Not severely malnourished (WLZ < −3 SD) or severely anemic	6	98% currently breast fed, 428, 86%	Animal vs. plant protein	WinFood Classic (WFC): provided 19 g of protein per 100 g dry weigh WinFood Lite (WFL): provided 14.6 g of protein per 100 g dry weight	Corn-soy blend plus (CSB+): provided 15.1 g of protein per 100 g dry weight	9	WLZ
Wall et al. [29], 2019, New Zealand and Australia, parallel group, double-blind	Healthy children 12 mo of age	12	Healthy, 160, 85%	High vs. low protein	GUMLi—standard cow milk-based product with reduced protein (1.7 g/100 mL) also fortified with micronutrients (including vitamin D and iron), probiotics, and prebiotics (i.e., a synbiotic) PER 15.9%	Whole pasteurized and homogenized cow milk (3.1 g/100 mL) PER = 17.51%	12	WLZ, FFM, FM (bioelectrical impedance)
Tang et al. [33], 2018, United States, parallel group, single-blind	Exclusively formula fed	5	Exclusively formula fed, 64, 85%	Meat vs. dairy	Isoproteic total protein intake during the intervention was targeted at 15% of total energy consumption or 3 g/kg/d	Meat group: (puréed meats) and dairy group: (infant yogurt, cheese, and whey protein powder)	7	WLZ, IGF-1, IGFBP-3
Agapova et al. [18], 2018, Malawi, cluster randomized, open-label	Children aged 12–23 mo living within walking distance of one of the village clusters	12–23	Non malnourished, human milk fed, 331, 99%	High vs. low protein	(Isocaloric) Common bean/cowpea flour: 23%–26% protein	Control (corn-soy blend): 13% protein	12	WLZ
Stephenson et al. [27], 2017, Malawi, cluster randomized, open-label	Children aged 12–23 mo living within walking distance of 1 of the village clusters	6	High prevalence of stunting and other forms of malnutrition, 291, 98%	High vs. low Protein	Cowpeas/common beans: PER: 12.9%–13.2%	Control: 10.7%	6	WLZ
Ochoa et al. [26], 2017, Peru, cluster randomized, single-blind	Children aged 6–36 mo who regularly participated (>4 times/wk) in either daycare centers or community nutritional centers	6–36	<1% MAM, 441, 99%	High vs. low protein	FPi diet: total protein 12.2 g (5.5 g animal protein and 6.8 g vegetable protein) Primary difference—protein quality The FPi group replaced 50% of the animal protein in the diet with fish protein isolate	Control diet: total protein 12.0 g (5.2 g animal protein and 6.8 g vegetable protein) and continued with standard animal protein sources (beef, chicken, pork, liver, and egg)	6	WLZ
Skau et al. [31], 2015, Cambodia, parallel group, single-blind	Infants aged 6 mo from villages in selected communes	6–15	40% of children were stunted, 11% were wasted, 419, 85.4%	Meat vs. dairy	WinFood: 474 kcal, 15.4g protein, 10.3 g fat; includes fish/spiders (14% ASF)	Corn-soy blend++: 458 kcal, 16.8 g protein, 10.7 g fat; includes skimmed milk (8%), soy	9	FFM FM (deuterium dilution) WLZ
Borg et al. [30], 2018, Cambodia, cluster randomized, open-label	Healthy singletons aged 6–11 mo	6–11	High prevalence of underweight and stunting, poor infant feeding practice, 485, 60%	High vs. low protein	RUSF: local ingredients, including small freshwater fish, soy, mung beans, and coconut Similar energy, protein, carbohydrate, and lipid content	Corn-soy blend (CSB) ++: milk or whey powder as the animal-source food	6	WLZ
Ghosh et al. [19], 2019, Ghana, cluster randomized, single-blind	Singleton term birth; exclusively or predominantly human milk fed	6–18	Exclusively or predominantly human milk fed, 792, 80%	High vs. low protein	KOKO Plus (KP): 2.9 g of protein per sachet, with high-quality protein and essential amino acids like lysine	Micronutrient powder (MN): only micronutrients without protein content	12	WLZ
Iannotti et al. [20], 2017, Ecuador, parallel group, single-blind	Infants aged 6–9 mo, singleton birth, in good health	6–9	Baseline prevalence of stunting was 38%, 163, 93%	High vs. low protein	Egg intervention group (1 egg/d for 6 mo)	Control group (no intervention)	6	BMIZ
Tang et al. [9], 2014, United States, parallel group, single-blind	Exclusively human milk fed infants, born at term (37–42 wk of gestation) with birth weight appropriate for gestational age	5	Exclusively human milk fed, 42, 100%	Animal vs. plant protein	Meat group: protein intake: commercially prepared puréed meats (71 g total per serving, equivalent to 8 g protein) as complementary food, aiming for 1–2 servings per day	Cereal group: Protein intake: iron- and zinc-fortified cereals with lower protein content compared with the meat group	6	WLZ, IGF-I, IGFBP3
Tang et al. [36], 2014, China, cluster randomized, open-label	Term delivery without serious neonatal complications and being exclusively human milk fed	6–18	30% stunting, 1471, >90%	Animal vs. plant protein	Meat group (35 g per 1216 kcal energy-overall) protein energy ratio:11.52%	Cereal group (29 g per 1162 kcal energy-overall) protein energy ratio: 9.98%	12	WLZ
Krebs et al. [34], 2012, Democratic Republic of Congo, Zambia, Guatemala, Pakistan, cluster randomized, open-label	Infants aged 3–4 mo who were primarily or exclusively human milk fed and whose mothers intended to continue human milk feeding for at least the first year	6–18	High rates of stunting, 1236, 86.1%	Animal vs. plant protein	Meat group: daily provision of lyophilized beef equivalent to 30–45 g cooked meat from 6 to 18 mo	Cereal group: micronutrient-fortified rice-soy cereal, equicaloric to meat portions (70 and 105 kcal/d)	12	WLZ
Long et al. [24], 2012, Kenya, parallel group, open-label	Toddlers aged 11–40 mo from the Embu District area	11–40	High stunting and underweight prevalence, 274, 70%	High vs. low protein	Meat porridge: 258 kcal, 13.0 g protein, 7.2g fat	Milk porridge: 255 kcal, 5.9 g protein, 8.4 g fat	5	WLZ
Larnkjær et al. [23], 2009, Denmark, parallel group, open-label	Healthy, singleton infants, with normal birth weight, no major pregnancy or birth complications, daily consumption of infant formula or whole milk	9–12	term, predominantly formula fed, 94, 88%	High vs. low protein	Whole milk group: higher protein content (22% protein energy percentage) compared with infant formula	Infant formula group: lower protein content (10% protein energy percentage)	3	IGF-1, IGFBP3
Tang et al. [32], 2019, United States, parallel group, single-blind	Exclusively formula fed	5	Exclusively formula fed, 64, 85%	Meat vs. dairy	Isoproteic total protein intake during the intervention was targeted at 15% of total energy consumption or 3 g/kg/d meat group: (puréed meats)	Dairy group: infant yogurt, cheese, and whey protein powder	7	WLZ, IGF-1, IGFBP-3
Mangani et al. [25], 2015, Malawi, parallel group, single-blind	Infants aged 5.5–6.5 mo	6–18	Human milk fed, 840, 89%	Animal vs. plant protein high vs. low protein	Comparison 1: milk-based LNS. Same protein energy and fat intake Comparison 2 Additional protein intake: CSB (with low fat):	Comparison 1: Soy-based LNS Comparison 2: No additional supplementation	12	WLZ
Mbabazi et al. [35], 2023, Uganda, parallel group, open-label	Children aged 6–24 mo with stunting	12–59	Stunted children, 1200, 95%	Animal vs. plant protein	Same calories, lipids, and proteins: milk vs. soy comparison milk protein + whey permeate	Soy protein + maltodextrin	3	WLZ, FFMI, FMI (bioelectrical impedance)

Abbreviations: ASF, Animal Source Foods; FPi, Fractional Protein intake; LNS, Lipid-based Nutrient Supplement; PER, Protein Efficiency Ratio; KOKO, Local maize-based porridge commonly used as complementary food in Ghana; MAM, Moderate Acute Malnutrition; BMIZ, Body Mass Index-for-Age Z-score; FFM, fat-free mass; FFMI, fat-free mass index; FM, fat mass; FMI, fat mass index; GUMLi, Growing Up Milk “Lite”; IGF-1, insulin-like growth factor-1; IGFBP-3, insulin-like growth factor binding protein-3; RDA, recommended dietary allowance; RUSF, ready-to-use supplementary food; WAZ, weight-for-age *Z*-score; WLZ, weight-for-length *Z*-score.

Most included studies were funded by government agencies, international organizations, or independent grant agencies. Only a few studies were supported by industry sources but were conducted in collaboration with public research institutions or the National Institute of Health.

### Risk of bias

Of the 12 studies included in the meta-analysis, the risk of bias was assessed using both the Cochrane Risk of Bias 2.0 (RoB 2.0) tool and the Cochrane Risk of Bias for Cluster Randomized Trials (Supplemental Figure 1a–d). Four studies were classified as having a low risk of bias [25,28,29,33]. Seven studies were categorized as having some concerns, primarily because of limited information or minor issues in certain domains, including uncertainties regarding randomization processes or adherence to intended interventions [18,21,24,31,[34], [35], [36]]. One study was considered to have a high risk of bias due to significant loss to follow-up [30].

### Meta-analysis results

#### High- and low-protein intake

**FIGURE 2:**
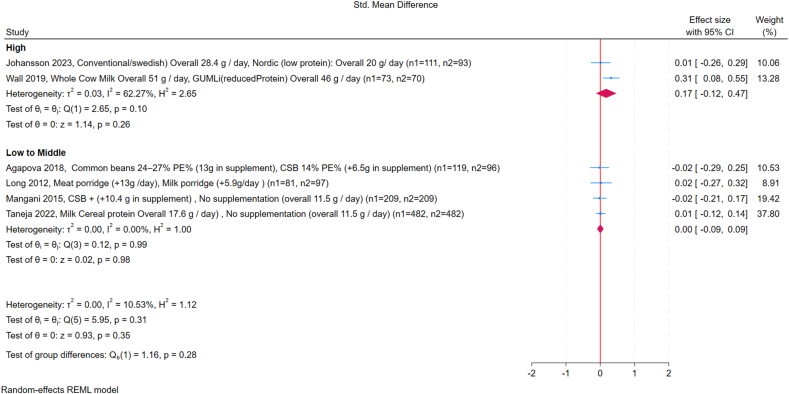
Forest plot of the effect of high compared with low protein supplementation on weight-for-length *Z*-score (WLZ), grouped by country income classification (low-to-middle-income compared with high-income, according to World Bank criteria). The outcome is expressed as the standardized mean difference (SMD). Effect sizes are presented as SMDs, with diamonds representing pooled summary estimates using a random-effects Restricted Maximum Likelihood (REML) model. “No supplementation” refers to groups receiving no additional protein beyond the habitual diet. GUMLi is (reduced protein) refers to a growing-up milk formulation with reduced protein content, used as a dietary intervention in young children. CI, confidence interval; CSB, corn-soy blend; GUMLi, Growing Up Milk “Lite”; PE%, protein energy percentage.

**FIGURE 3:**
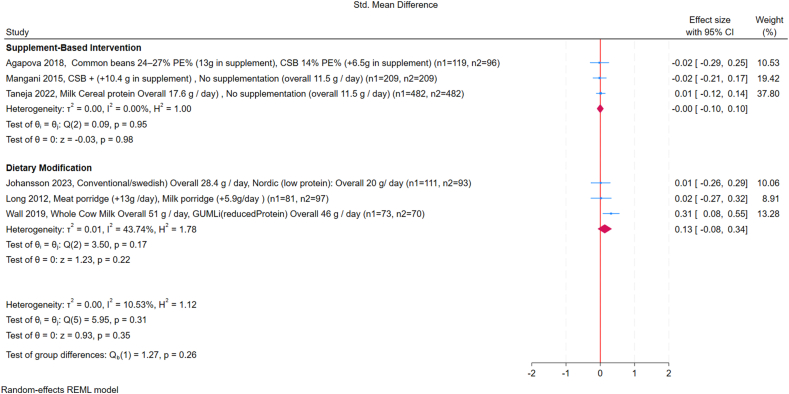
Forest plot of the effect of high compared with low protein supplementation on weight-for-length *Z*-score (WLZ), grouped by supplement type (supplement-based intervention compared with dietary modification). The outcome is expressed as the standardized mean difference (SMD). Effect sizes are presented as SMDs, with diamonds representing pooled summary estimates using a random-effects REML model. “No supplementation” refers to groups receiving no additional protein beyond the habitual diet. GUMLi is (reduced Protein) refers to a growing-up milk formulation with reduced protein content, used as a dietary intervention in young children. CI, confidence interval; CSB, corn-soy blend; GUMLi, Growing Up Milk “Lite”; PE%, protein energy percentage.

**FIGURE 4:**
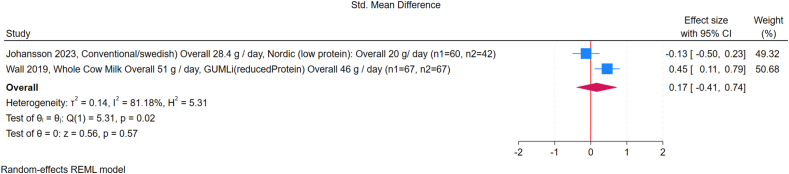
Forest plot of the effect of high compared with low protein supplementation on fat mass (FM). Effect sizes are presented as standardized mean differences (SMDs), with diamonds representing pooled summary estimates using a random-effects REML model. CI, confidence interval.

**FIGURE 5:**
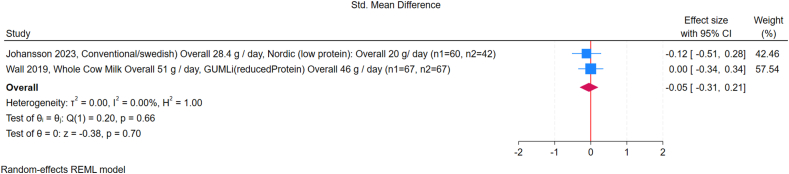
Forest plot of the effect of high compared with low protein intake and fat-free mass (FFM) in children, grouped by supplement type (supplement-based intervention compared with dietary modification). GUMLi is (reduced Protein) refers to a growing-up milk formulation with reduced protein content, used as a dietary intervention in young children. CI, confidence interval; GUMLi, Growing Up Milk “Lite.”

Across the included studies, subgroup analyses showed no significant differences in WLZ between high- and low-protein interventions. Subgroup analysis by country income classification showed no significant differences: in high-income countries, the SMD was 0.17 (95% CI: –0.12, 0.47; *I*^2^ = 62%), whereas in LMICs, the SMD was 0.0 (95% CI: –0.09, 0.09; *I*^2^ = 0%) (Figure 2) [18,21,24,25,28,29]. For supplement-based interventions, the pooled SMD was 0.0 (95% CI: –0.10, 0.10; *I*^2^ = 0%); for dietary modification studies, the pooled SMD was 0.13 (95% CI: –0.08, 0.34; *I*^2^ = 43%) (Figure 3). No significant effects were observed for FM or FFM in any of the studies included for quantitative synthesis (FIGURE 4, FIGURE 5). Study by Manapurath et al. [37], the only low-income setting study, was not included in the plot but likewise reported no differences at 12 mo. Data on insulin and IGF-1 were limited; only 1 study per comparison reported these outcomes, precluding quantitative synthesis. Results are described narratively in the Supplementary data.

#### Meat and dairy supplementation

**FIGURE 6:**
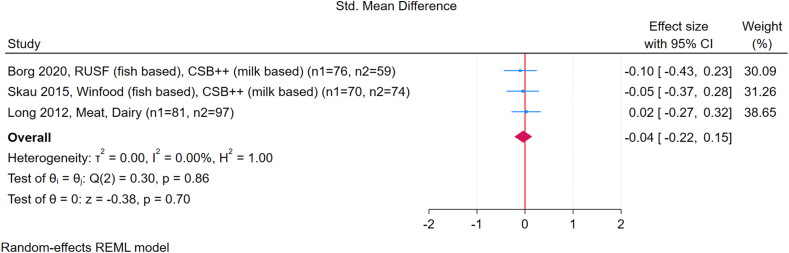
Forest plot comparing the effect of meat-based and dairy-based protein intake during complementary feeding on weight-for-length *Z*-score (WLZ). In these studies, dairy-based groups received corn-soy blend plus plus (CSB++), a fortified corn-soy blend containing 8% dried skimmed milk as the dairy-source ingredient. Meat- or fish-based groups received RUSF (ready-to-use supplementary food, fish based), WinFood (complementary food with fish and edible spiders), or meat as the primary animal-source protein. Control. Effect sizes are presented as standardized mean differences (SMDs), with diamonds representing the pooled summary estimate and horizontal lines showing 95% confidence intervals. Sample sizes for each group are indicated in parentheses after the study name. Results were calculated using a random-effects REML model. GUMLi is (reduced Protein) refers to a growing-up milk formulation with reduced protein content, used as a dietary intervention in young children. CI, confidence interval; CSB, corn-soy blend; GUMLi, Growing Up Milk “Lite.”

Three studies comparing meat and dairy-based protein reported a pooled SMD of –0.04 WLZ scores (95% CI: –0.22, 0.15; *I*^2^ = 0.0%) (Figure 6) [24,30,31]. No specific data on FM, FFM, or metabolic outcomes were reported in these 3 trials. Tang et al. [33], the only study conducted in a high-income setting, was not included in the plot but showed that dairy protein was associated with higher weight gain at 12 mo. However, this effect was not sustained at 24 mo (not shown in the plot because of the unavailability of numerical values for this time point). As a sensitivity analysis, we excluded the study with a high risk of bias. The results were similar, with no significant difference in WLZ between groups (Supplemental Figure 2).

#### Animal and plant protein supplementation

**FIGURE 7:**
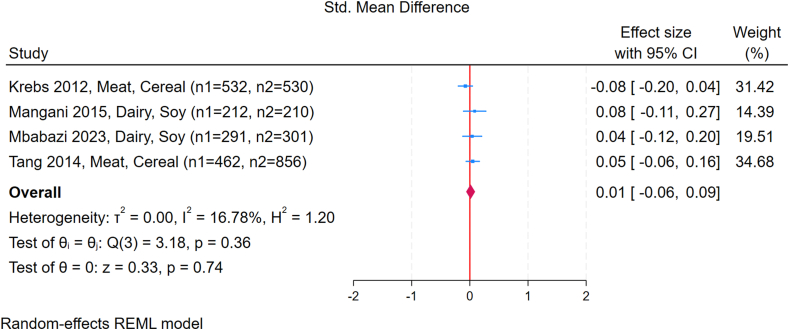
Forest plot of the effect of animal-based compared with plant-based protein intake during complementary feeding on weight-for-length *Z*-score (WLZ). Effect sizes are presented as standardized mean differences (SMDs), with diamonds representing the pooled summary estimate and horizontal lines showing 95% confidence intervals. Sample sizes for each group are indicated in parentheses after the study name. Results were calculated using a random-effects REML model. CI, confidence interval.

Four studies compared animal-based protein and plant-based protein reported a pooled SMD of 0.01 WLZ scores (95% CI: –0.06, 0.09; *I*^2^ = 17%) (Figure 7) [25,[34], [35], [36]]. No specific data on FM, FFM, or metabolic outcomes were reported in these 4 trials.

GRADE evidence for these outcomes is summarized in Supplemental Tables 4–6. The certainty of evidence across outcomes was low to very low, primarily because of concerns about risk of bias, heterogeneity, and imprecision. High compared with low protein supplementation showed no consistent effect on growth, body composition, or metabolic markers, with evidence downgraded due to variability in study populations and outcome measures. Similarly, animal compared with plant protein comparisons did not demonstrate meaningful differences in growth or adiposity, with some concerns related to indirectness. Meat compared with dairy protein comparisons showed variability in weight gain patterns, but findings were inconsistent across studies, with evidence downgraded due to high heterogeneity.

## Discussion

This systematic review and meta-analysis evaluated the effects of protein intake during complementary feeding on growth, body composition, and metabolic outcomes in young children. The findings indicate that higher protein intake did not affect WLZ, FM, or FFM across studies. There was insufficient evidence to determine whether higher protein intake during complementary feeding affects insulin or IGF-1 concentrations. The comparison between animal and plant proteins revealed no significant or meaningful differences in WLZ, suggesting that animal-source protein does not confer additional benefits for growth during complementary feeding [38]. Meat and dairy protein comparisons demonstrated some variability, without clear trends.

In exploring body composition more specifically, few studies assessed the effects of protein intake on FM. In low- and middle-income settings, Manapurath et al. [37] found no impact of higher protein supplementation on FM, likely because protein supports growth rather than fat deposition in populations with lower baseline intake. However, findings from high-income settings were inconsistent, whereas Wall et al. [29] observed higher FM with high-protein (whole cow milk) diets compared with a reduced-protein modified milk formula, Johansson et al. [21] found no significant differences in FM between a protein-reduced Nordic diet and a conventional Swedish diet. Taken together, the findings indicate that reducing early-life animal protein intake, whether through modifying milk composition or lowering overall protein intake, may help regulate fat accumulation in populations with adequate nutrition. Conversely, increasing protein intake in populations with lower dietary protein intake appears safe and beneficial. Owing to the small number of studies reporting insulin or IGF-1, no quantitative synthesis was possible, and the relationship between protein intake, growth factors, and adiposity could not be evaluated.

Systematic reviews indicate the influence of protein intake during early childhood on growth and metabolic outcomes, with varying results. Ferré et al. [39] (1 RCT and 2 cohort studies) found that higher protein intake during the second year of life was associated with increased FM at ages 2 and 7 y. Similarly, a Cochrane review by Gonzalez-Garay et al. [5] reported low-quality evidence for short-term impacts of high-protein formulas (>2.5 g/100 Kcal) on growth deficits during infancy, although some studies suggested a later increase in BMI. Arnesen et al. [40] in a meta-analysis (5 RCTs and 16 cohort studies) found a probable association between higher total and animal protein intake and higher BMI during early childhood (0.06 kg/m^2^ per 1% increase in protein energy). However, the evidence comparing animal and plant protein sources was inconclusive, as there were fewer studies [40]. In addition to these reviews, guidelines such as the Nordic Nutrition Recommendations suggest that animal proteins, particularly dairy, were associated with higher growth because of their higher leucine content and effects on IGF-1 stimulation [41]. Evidence from trials in formula-fed infants during early infancy shows that high-protein intake has been strongly associated with accelerated weight gain and adiposity [8,42]. Although these trials have informed early-life protein intake recommendations, their relevance to the complementary feeding period remains uncertain, as protein intake during this stage occurs within a more diverse diet. These findings, combined with the trends observed in this review, underscore the importance of understanding protein source and quantity in complementary feeding to balance growth promotion and metabolic health.

Current guidelines, such as those from the WHO and the European Society for Pediatrics Gastroenterology, Hepatology, and Nutrition, emphasize the importance of adequate protein intake during complementary feeding to support growth and development [43,44]. Although these guidelines provide general protein intake recommendations, they do not provide specific thresholds for protein quantity or detailed recommendations on the relative benefits of animal- compared with plant-based proteins. Our findings provide evidence that protein-enriched diets during complementary feeding do not increase adiposity or alter growth indices such as WLZ. However, the effects may depend on the dietary context, as seen in the variation between studies. Additionally, our comparison of animal- and plant-based proteins suggests no significant difference in their short-term effects on growth. The limited number of studies and variability in intervention designs may have contributed to the lack of detectable differences.

Limitations of this review include variability in intervention designs, protein sources, and baseline nutritional status, contributing to heterogeneity in the results. Surrogate measures such as WLZ limit the assessment of long-term impacts on adiposity. None of the included studies directly reported obesity outcomes. Additionally, our findings are limited by the short duration of follow-up in most studies, precluding conclusions about the impact of early protein supplementation on obesity or metabolic risk beyond the age of 2 y. Although the complementary feeding period (6–23 mo) is widely recognized as a critical window for child nutrition and development, the available studies do not allow us to determine whether specific ages within this period are more sensitive to protein intake with respect to growth or long-term metabolic outcomes. It is also possible that individual studies were underpowered to detect small effects, or that true effects of protein intake are not detectable at this age or within the range of protein concentrations studied.

Another limitation is the inconsistent reporting of dietary intake across studies. Nutritional intervention studies should consistently include baseline dietary recall data alongside the absolute difference in macronutrient intake to improve comparability and better assess the effects of protein intake on growth and body composition. By focusing on growth metrics such as WLZ, this review provides insights into protein’s role in growth, but cannot fully evaluate its influence on obesity risk.

Strengths of this review include its focus on RCTs, ensuring robust causal evidence, and its comprehensive assessment of growth, body composition, and metabolic outcomes. By including diverse settings, it highlights context-specific effects of protein interventions during the critical complementary feeding period. There was no discernible pattern of study findings according to funding source, and thus, funding-related bias is unlikely to have influenced the overall results.

In conclusion, this systematic review and meta-analysis found insufficient evidence to conclude that higher protein intake during complementary feeding consistently affects growth or body composition outcomes. Subgroup analyses showed no meaningful differences between dietary modification and supplement-based interventions, and comparisons between animal- and plant-based proteins revealed no significant effects. There was insufficient evidence to determine whether higher protein intake during complementary feeding affects insulin or IGF-1 concentrations. Given the heterogeneity of contexts and baseline nutritional status across studies, further evidence is needed to clarify whether specific dietary guidance should be tailored to context or population subgroups.

## Author contributions

The authors’ responsibilities were as follows – RM: conceptualized the study with key inputs from TAS and CPD, conducted the literature search, data extraction, and analysis, and drafted the manuscript with inputs from all authors; TAS, CPD: contributed to study screening decisions and provided critical oversight on methodology and interpretation; RC, ST, NB: provided inputs in study selection, review methods, and contributed to the manuscript draft; and all authors: reviewed, revised, and approved the final manuscript.

## Data availability

Data described in the article are publicly and freely available online.

## Funding

The review received funding from Centre for Intervention Science in Maternal and Child Health (CISMAC), Norway (2024 Data analysis/systematic review/meta-analysis/Methods paper grant). The funding agency had no role in designing the study; data collection, analysis, or interpretation of the results, in the preparation of the manuscript; or in the decision to submit for publication.

## Conflict of interest

CPD was supported in part by P30 DK040561. CPD is editor-in-chief of the *AJCN* but played no role in the editorial handing of this manuscript.

## References

[1] World Health Organization FaAOotUN, United Nations University (2007).

[2] Putet G., Labaune J.-M., Mace K., Steenhout P., Grathwohl D., Raverot V. (2016). Effect of dietary protein on plasma insulin-like growth factor-1, growth, and body composition in healthy term infants: A randomised, double-blind, controlled trial (Early Protein and Obesity in Childhood (EPOCH) study). Br. J. Nutr..

[3] Millward D.J. (2017). Nutrition, infection and stunting: The roles of deficiencies of individual nutrients and foods, and of inflammation, as determinants of reduced linear growth of children. Nutr. Res. Rev..

[4] Arsenault J.E., Brown K.H. (2017). Effects of protein or amino-acid supplementation on the physical growth of young children in low-income countries. Nutr. Rev..

[5] Gonzalez-Garay A.G., Serralde-Zúñiga A.E., Medina Vera I., Velasco Hidalgo L., Alonso Ocaña M.V. (2023). Higher versus lower protein intake in formula-fed term infants. Cochran. Database Syst. Rev..

[6] Totzauer M., Escribano J., Closa-Monasterolo R., Luque V., Verduci E., ReDionigi A. (2022). Different protein intake in the first year and its effects on adiposity rebound and obesity throughout childhood: 11 years follow-up of a randomized controlled trial, Pediatr. Obes.

[7] Agostoni C., Scaglioni S., Ghisleni D., Verduci E., Giovannini M., Riva E. (2005). How much protein is safe?. Int. J. Obes..

[8] Koletzko B., Demmelmair H., Grote V., Prell C., Weber M. (2016). High protein intake in young children and increased weight gain and obesity risk. Am. J. Clin. Nutr..

[9] Tang M., Krebs N.F. (2014). High protein intake from meat as complementary food increases growth but not adiposity in breastfed infants: A randomized trial. Am. J. Clin. Nutr..

[10] Institute of Medicine (2005). Standing Committee on the Scientific Evaluation of Dietary Reference I, Dietary Reference Intakes for Energy, Carbohydrate, Fiber, Fat, Fatty Acids, Cholesterol, Protein, and Amino Acids.

[11] Organization W.H. (2007).

[12] World Health Organization (2006).

[13] (2024). Covidence Systematic Review Software [Internet].

[14] Borenstein M., Julian L., Higgins P.T., Hannah R. (2009).

[15] T Higgins J.P., Thomas J., Chandler J., Cumpston M., Li T., Page M.J., Welch V.A. (2024). Cochrane handbook for systematic reviews of interventions version 6.5.

[16] Sterne J.A.C., Savović J., Page M.J., Elbers R.G., Blencowe N.S., Boutron I. (2019). RoB 2: A revised tool for assessing risk of bias in randomised trials. BMJ.

[17] Balshem H., Helfand M., Schünemann H.J., Oxman A.D., Kunz R., Brozek J. (2011). GRADE guidelines: 3. Rating the quality of evidence. J. Clin. Epidemiol..

[18] Agapova S.E., Stephenson K.B., Divala O., Kaimila Y., Maleta K.M., Thakwalakwa C. (2018). Additional common bean in the diet of Malawian children does not affect linear growth, but reduces intestinal permeability. J. Nutr..

[19] Ghosh S.A., Strutt N.R., Otoo G.E., Suri D.J., Ankrah J., Johnson T. (2019). A macro- and micronutrient-fortified complementary food supplement reduced acute infection, improved haemoglobin and showed a dose–response effect in improving linear growth: A 12-month cluster randomised trial. J. Nutr. Sci..

[20] Iannotti L.L., Lutter C.K., Stewart C.P., Gallegos Riofrío C.A., Malo C., Reinhart G. (2017). Eggs in early complementary feeding and child growth: A randomized controlled trial. Pediatrics.

[21] Johansson U., Öhlund I., Lindberg L., Hernell O., Lönnerdal B., Venables M. (2023). A randomized, controlled trial of a Nordic, protein-reduced complementary diet in infants: Effects on body composition, growth, biomarkers, and dietary intake at 12 and 18 months. Am. J. Clin. Nutr..

[22] Konyole S.O., Omollo S.A., Kinyuru J.N., Skau J., Owuor B.O., Estambale B.B. (2019). Effect of locally produced complementary foods on fat-free mass, linear growth, and iron status among Kenyan infants: A randomized controlled trial, Matern. Child Nutr..

[23] Larnkjær A., Hoppe C., Mølgaard C., Michaelsen K.F. (2009). The effects of whole milk and infant formula on growth and IGF-I in late infancy. Eur. J. Clin. Nutr..

[24] Long J.K., Murphy S.P., Weiss R.E., Nyerere S., Bwibo N.O., Neumann C.G. (2012). Meat and milk intakes and toddler growth: A comparison feeding intervention of animal-source foods in rural Kenya. Public Health Nutr.

[25] Mangani C., Maleta K., Phuka J., Cheung Y.B., Thakwalakwa C., Dewey K. (2015). Effect of complementary feeding with lipid-based nutrient supplements and corn-soy blend on the incidence of stunting and linear growth among 6- to 18-month-old infants and children in rural Malawi. Matern. Child Nutr..

[26] Ochoa T.J., Baiocchi N., Valdiviezo G., Bullon V., Campos M., Llanos-Cuentas A. (2017). Evaluation of the efficacy, safety and acceptability of a fish protein isolate in the nutrition of children under 36 months of age. Public Health Nutr.

[27] Stephenson K.B., Agapova S.E., Divala O., Kaimila Y., Maleta K.M., Thakwalakwa C. (2017). Complementary feeding with cowpea reduces growth faltering in rural Malawian infants: A blind, randomized controlled clinical trial. Am. J. Clin. Nutr..

[28] Taneja S., Upadhyay R.P., Chowdhury R., Kurpad A.V., Bhardwaj H., Kumar T. (2022). Impact of supplementation with milk–cereal mix during 6–12 months of age on growth at 12 months: A 3-arm randomized controlled trial in Delhi, India. Am. J. Clin. Nutr..

[29] Wall C.R., Hill R.J., Lovell A.L., Matsuyama M., Milne T., Grant C.C. (2019). A multicenter, double-blind, randomized, placebo-controlled trial to evaluate the effect of consuming Growing Up Milk “Lite” on body composition in children aged 12–23 mo. Am. J. Clin. Nutr..

[30] Borg B., Mihrshahi S., Griffin M., Sok D., Chhoun C., Laillou A. (2018). Randomised controlled trial to test the effectiveness of a locally-produced ready-to-use supplementary food (RUSF) in preventing growth faltering and improving micronutrient status for children under two years in Cambodia: A study protocol. Nutr. J..

[31] Skau J.K., Touch B., Chhoun C., Chea M., Unni U.S., Makurat J. (2015). Effects of animal source food and micronutrient fortification in complementary food products on body composition, iron status, and linear growth: A randomized trial in Cambodia. Am. J. Clin. Nutr..

[32] Tang M., Andersen V., Hendricks A.E., Krebs N.F. (2019). Different growth patterns persist at 24 months of age in formula-fed infants randomized to consume a meat- or dairy-based complementary diet from 5 to 12 months of age. J. Pediatr..

[33] Tang M., Hendricks A.E., Krebs N.F. (2018). A meat- or dairy-based complementary diet leads to distinct growth patterns in formula-fed infants: A randomized controlled trial. Am. J. Clin. Nutr..

[34] Krebs N.F., Mazariegos M., Chomba E., Sami N., Pasha O., Tshefu A. (2012). Randomized controlled trial of meat compared with multimicronutrient-fortified cereal in infants and toddlers with high stunting rates in diverse settings. Am. J. Clin. Nutr..

[35] Mbabazi J., Pesu H., Mutumba R., Filteau S., Lewis J.I., Wells J.C. (2023). Effect of milk protein and whey permeate in large quantity lipid-based nutrient supplement on linear growth and body composition among stunted children: A randomized 2 × 2 factorial trial in Uganda. PLoS Med.

[36] Tang M., Sheng X.-Y., Krebs N.F., Hambidge K.M. (2014). Meat as complementary food for older breastfed infants and toddlers: A randomized, controlled trial in rural China. Food Nutr. Bull..

[37] Manapurath R., Chowdhury R., Upadhyay R.P., Kurpad A.V., Bose B., Devi S. (2025). Impact of high- and moderate-protein supplementation on early-life obesity and body composition: A randomized controlled trial in India. Am. J. Clin. Nutr..

[38] Vissamsetti N., Simon-Collins M., Lin S., Bandyopadhyay S., Kuriyan R., Sybesma W. (2024). Local sources of protein in low- and middle-income countries: How to improve the protein quality?. Curr. Dev. Nutr..

[39] Ferré N., Luque V., Closa-Monasterolo R., Zaragoza-Jordana M., Gispert-Llauradó M., Grote V. (2021). Association of protein intake during the second year of life with weight gain-related outcomes in childhood: A systematic review. Nutrients.

[40] Arnesen E.K., Thorisdottir B., Lamberg-Allardt C., Bärebring L., Nwaru B., Dierkes J. (2022). Protein intake in children and growth and risk of overweight or obesity: A systematic review and meta-analysis. Food Nutr. Res..

[41] Hörnell A., Lagström H., Lande B., Thorsdottir I. (2013). Protein intake from 0 to 18 years of age and its relation to health: A systematic literature review for the 5th Nordic Nutrition Recommendations. Food Nutr. Res..

[42] Koletzko B., von Kries R., Closa R., Escribano J., Scaglioni S., Giovannini M. (2009). Lower protein in infant formula is associated with lower weight up to age 2 y: A randomized clinical trial. Am. J. Clin. Nutr..

[43] World Health Organization (2023). https://www.who.int/publications/i/item/9789240081864.

[44] Fewtrell M., Bronsky J., Campoy C., Domellöf M., Embleton N., Fidler Mis N. (2017). Complementary feeding: A position paper by the European Society for Paediatric Gastroenterology, Hepatology, and Nutrition (ESPGHAN) Committee on Nutrition. J. Pediatr. Gastroenterol. Nutr..

