# Supplementary material for: Effect of early-life protein supplementation on childhood obesity and related metabolic outcomes: a systematic review and meta-analysis
Source: Am J Clin Nutr. 2025 Sep 13;122(6):1797–808. doi: 10.1016/j.ajcnut.2025.09.017 (PMC12799402; doi:10.1016/j.ajcnut.2025.09.017)
Supplement: Multimedia component 1 [file mmc1.docx]

# Table S1. Search Strategy Used for Each Database

# **(dated 31/05/2024)**

| **Protein** | **AND** | **Obesity / Body Composition** | **NOT** | **Exclusion** |
| --- | --- | --- | --- | --- |
| ((“artificial milk”[Tw] OR “formula milk”[Tw] OR "synthetic milk" OR "milk substitute" OR "infant feeding"[Tw] OR "baby food"[Tw] OR "infant formula"[Tw] OR "complementary feeding"[Tw] OR "complementary food"[Tw] OR "weaning"[Tw] OR "infant nutrition"[Tw] OR "Infant Formula"[Mesh] OR "follow-on formula"[Tw] OR "toddler formula"[Tw] OR "homemade food"[Tw] OR "Dietary Supplements"[MAJR] OR “Infant Nutritional Physiological Phenomena”[Mesh] OR "Child Nutritional Physiological Phenomena"[MAJR]) **AND** ("proteins"[MeSH] OR "Protein source"[Tw] OR "proteins"[MeSH] OR "Protein source"[Tw] OR "Dietary Proteins"[Mesh] OR "Protein-Rich Food"[Tw] OR "Protein-Energy Malnutrition"[MeSH] OR “Protein Intake”[Tw] OR “Protein Consumption”[Tw] OR “Protein Requirement”[Tw] OR “Protein Assessment”[Tw] OR "Dietary Proteins/administration and dosage"[MeSH] OR "protein energy ratio"[Title/Abstract])) |  | ("BMI-for-age Z score"[Tw] OR "Pediatric Obesity/genetics"[MAJR] OR "Overweight"[Mesh] OR "Body composition"[Tw] OR "adiposity"[Tw] OR "obes*"[MeSH Terms] OR skinfold[Tw] OR Distribution, Body Fat[Mesh] OR "Body Fat Patter*"[Tw] OR "Body fat" [TiAb] OR "catchup growth"[Tw] OR “Growth Disorders”[Mesh] OR “Stunting”[Tw] OR "Anthropometry"[Tw] OR "weight gain"[All Fields] OR "Body Weight"[MeSH])) |  | "mice"[Title/Abstract] OR "rats"[Title/Abstract] OR ("lobster"[Title/Abstract] OR ("hamster"[Title/Abstract] |

# Hit results

| **Search** | **Results** |
| --- | --- |
| "BMI-for-age Z score"[Tw] OR "Pediatric Obesity/genetics"[MAJR] OR "Overweight"[Mesh] OR "Body composition"[Tw] OR "adiposity"[Tw] OR "obes*"[MeSH Terms] OR skinfold[Tw] OR Distribution, Body Fat[Mesh] OR "Body Fat Patter*"[Tw] OR "Body fat" [TiAb] OR "catchup growth"[Tw] OR "Growth Disorders"[Mesh] OR "Stunting"[Tw] OR "Anthropometry"[Tw] OR "weight gain"[All Fields] OR "Body Weight"[MeSH] | 7,19,068 |
| (("artificial milk"[Tw] OR "formula milk"[Tw] OR "synthetic milk" OR "milk substitute" OR "infant feeding"[Tw] OR "baby food"[Tw] OR "infant formula"[Tw] OR "complementary feeding"[Tw] OR "complementary food"[Tw] OR "weaning"[Tw] OR "infant nutrition"[Tw] OR "Infant Formula"[Mesh] OR "follow-on formula"[Tw] OR "toddler formula"[Tw] OR "homemade food"[Tw] OR "Dietary Supplements"[MAJR] OR "Infant Nutritional Physiological Phenomena"[Mesh] OR "Child Nutritional Physiological Phenomena"[MAJR]) **AND** ("proteins"[MeSH] OR "Protein source"[Tw] OR "proteins"[MeSH] OR "Protein source"[Tw] OR "Dietary Proteins"[Mesh] OR "Protein-Rich Food"[Tw] OR "Protein-Energy Malnutrition"[MeSH] OR "Protein Intake"[Tw] OR "Protein Consumption"[Tw] OR "Protein Requirement"[Tw] OR "Protein Assessment"[Tw] OR "Dietary Proteins/administration and dosage"[MeSH] OR "protein energy ratio"[Title/Abstract])) | 31,249 |
| "mice"[Title/Abstract] OR "rats"[Title/Abstract] OR "lobster"[Title/Abstract] OR "hamster"[Title/Abstract] | 20,42,668 |
| **COMBINED** | **3,562** |

**EMBASE**

1 (BMI-for-age Z score or Pediatric Obesity or Overweight or Body composition or adiposity obes* or skinfold).tw. or Body Fat.af. or catchup growth.tw. or Growth Disorders.tw. or Stunting.tw. or anthropometry.tw. or weight gain.tw. or Body Weight.tw. 851328

2 limit 1 to yr="2000 - 2024" 720158

3 ((artificial milk or formula milk or synthetic milk or milk substitute or infant feeding or baby food).tw. or infant formula.af. or complementary feeding.tw. or complementary food.tw. or weaning.tw. or infant nutrition.tw. or Infant Formula.tw. or follow-on formula.tw. or toddler formula.tw. or homemade food.tw. or Dietary Supplements.tw.) and (proteins or Protein source or Dietary Proteins or Protein-Rich Food or Protein-Energy Malnutrition or Protein Intake or Protein Consumption or Protein Requirement or Protein Assessment or protein energy ratio).tw. 6776

4 limit 3 to yr="2000 - 2024" 5926

5 2 and 4 = 1878

**Web of Science (Core Collection; searched 31 May 2024)**

TS= ("artificial milk" OR "formula milk" OR "synthetic milk" OR "milk substitute" OR "infant feeding" OR "baby food" OR "infant formula" OR "complementary feeding" OR "complementary food" OR "weaning" OR "infant nutrition" OR "follow-on formula" OR "toddler formula" OR "homemade food" OR "dietary supplements")

AND

TS= ("proteins" OR "protein source" OR "dietary proteins" OR "protein-rich food" OR "protein-energy malnutrition" OR "protein intake" OR "protein consumption" OR "protein requirement" OR "protein assessment" OR "protein energy ratio")

AND

TS= ("BMI-for-age Z score" OR "pediatric obesity" OR "overweight" OR "body composition" OR "adiposity" OR "obesity" OR "skinfold" OR "body fat" OR "catchup growth" OR "growth disorders" OR "stunting" OR "anthropometry" OR "weight gain" OR "body weight")

Limits: Humans, 2000–2024, English

Results: 301 records

**CINAHL (EBSCOhost; searched May 2024)**

S1: (MH "Infant Formula" OR MH "Infant Nutrition" OR "infant formula" OR "complementary feeding" OR "complementary food" OR "weaning" OR "infant feeding" OR "dietary supplements") AND (MH "Dietary Proteins" OR "protein intake" OR "dietary protein" OR "protein supplement" OR "protein source")

Results: 1,919

S2: (MH "Body Composition" OR MH "Obesity" OR "BMI" OR "body fat" OR "overweight" OR "weight gain" OR "stunting" OR "anthropometry")

Results: 218,649

S3: S1 AND S2

Limiters: Publication date: Jan 1, 2000–May 31, 2024; Exclude MEDLINE records; Age Groups: Infant (1–23 months), Child, Preschool (2–5 years); English

Results: 40

**Cochrane Library (searched May 31, 2025)**

Search strategy:

#1:

("Dietary Protein"):ti,ab,kw

Results: 1,323

#2:

("artificial milk" OR "formula milk" OR "synthetic milk" OR "milk substitute" OR "infant feeding" OR "baby food" OR "infant formula" OR "complementary feeding" OR "complementary food" OR "weaning" OR "infant nutrition" OR "follow-on formula" OR "toddler formula" OR "homemade food" OR "dietary supplements"):ti,ab,kw

Results: 25,262

#3:

#1 AND #2 in Cochrane Reviews, Cochrane Protocols, Trials, Clinical Answers, Editorials, Special Collections

Results: 114

#4:

("BMI-for-age Z score" OR "overweight" OR "body composition" OR "adiposity" OR "stunting" OR "weight gain"):ti,ab,kw

Limits: Cochrane Library publication date between Jan 2000 and May 2024;

in Cochrane Reviews, Cochrane Protocols, Trials, Clinical Answers, Editorials, Special Collections

Results: 48,474

#5:

#3 AND #4

Limits: Cochrane Library publication date between Jan 2000 and May 2024

Results: 31

**Table S2: List of Excluded Studies**

| **Study** | **Title** | **Reasons** |
| --- | --- | --- |
| Ackatia-Armah 2015 | Malian children with moderate acute malnutrition who are treated with lipid-based dietary supplements have greater weight gains and recovery rates than those treated with locally produced cereal-legume products: a community-based, cluster-randomized trial | Author contacted, no response |
| Adams 2020 | The impact of maternal supplementation during pregnancy and the first 6 months postpartum on the growth status of the next child born after the intervention period: Follow-up results from Bangladesh and Ghana | LNS Supplement |
| Adu-Afarwuah 2007 | Randomized comparison of 3 types of micronutrient supplements for home fortification of complementary foods in Ghana: effects on growth and motor development23 | Comparison of micronutrients between groups |
| Adu-Afarwuah 2016 | Small-quantity, lipid-based nutrient supplements provided to women during pregnancy and 6 mo postpartum and to their infants from 6 mo of age increase the mean attained length of 18-mo-old children in semi-urban Ghana: a randomized controlled trial | the comparison groups (IFA and MMN) received micronutrients during pregnancy |
| Allman 2020 | Dietary Protein Intake during Pregnancy Is Not Associated with Offspring Insulin Sensitivity during the First Two Years of Life. | Observational study |
| Amoah 2024 | Factors Contributing to Malnutrition among Children Under 5â€‰Years at St. Elizabeth Catholic Hospital, Ahafo Hwidiem. | Observational study |
| Andersen 2011 | A randomized controlled intervention with fish oil versus sunflower oil from 9 to 18 months of age: exploring changes in growth and skinfold thicknesses | ntervention is focused on long-chain polyunsaturated fatty acids (LCPUFAs) from fish oil and sunflower oil) |
| Anshori 2020 | Relationship energy and protein intake with the incidence of stunting among toddler aged (25-60 months) in Mangkung Village, District of central Lombok | Observational study |
| Araya 1995 | [Effect of protein and carbohydrate preloads on food and energy intakes in preschool children with different nutritional status] | Observational study |
| Arimond 2017 | Impact of small quantity lipid-based nutrient supplements on infant and young child feeding practices at 18 months of age: results from four randomized controlled trials in Africa. | LNS with fortified MNS- No group for comparison |
| Arnesen 2022 | Protein intake in children and growth and risk of overweight or obesity: A systematic review and meta-analysis | Review article |
| Arsenault 2017 | Effects of protein or amino-acid supplementation on the physical growth of young children in low-income countries. | Review article |
| Arsenault 2017 | Dietary Protein Intake in Young Children in Selected Low-Income Countries Is Generally Adequate in Relation to Estimated Requirements for Healthy Children, Except When Complementary Food Intake Is Low. | Review article |
| Asare 2022 | Animal-source foods as a suitable complementary food for improved physical growth in 6 to 24-month-old children in low- and middle-income countries: a systematic review and meta-analysis of randomised controlled trials. | Review article |
| Ashorn 2015 | Supplementation of Maternal Diets during Pregnancy and for 6 Months Postpartum and Infant Diets Thereafter with Small-Quantity Lipid-Based Nutrient Supplements Does Not Promote Child Growth by 18 Months of Age in Rural Malawi: A Randomized Controlled Trial | LNS from pregnancy to childhood in one group (no comparison possible) |
| Axelsson 1987 | Protein and energy intake during weaning: I. Effects on growth | Study conducted in 1980's |
| Baye 2013 | Nutrient intakes from complementary foods consumed by young children (aged 12-23 months) from North Wollo, northern Ethiopia: the need for agro-ecologically adapted interventions. | Observational study |
| Becquey 2019 | Impact on child acute malnutrition of integrating a preventive nutrition package into facility-based screening for acute malnutrition during well-baby consultation: A cluster-randomized controlled trial in Burkina Faso | LNS with fortified MNS- No group for comparison |
| Beyerlein 2017 | Intake of Energy and Protein is Associated with Overweight Risk at Age 5.5 Years: Results from the Prospective TEDDY Study. | Observational study |
| Billah 2017 | A community-based cluster randomised controlled trial to evaluate the effectiveness of different bundles of nutrition-specific interventions in improving mean length-for-age z score among children at 24Â months of age in rural Bangladesh: study protocol. | Ongoing study (Protocol) |
| Billeaud 2022 | A new partially hydrolyzed whey-based follow-on formula with age-adapted protein content supports healthy growth during the first year of life | Interventions started after birth |
| Borg 2018 | Randomised controlled trial to test the effectiveness of a locally-produced ready-to-use supplementary food (RUSF) in preventing growth faltering and improving micronutrient status for children under two years in Cambodia: a study protocol. | Ongoing study (Protocol) |
| Borschel 2014 | Growth of healthy term infants fed ready-to-feed and powdered forms of an extensively hydrolyzed casein-based infant formula: a randomized, blinded, controlled trial | Less than 6 months |
| Brands 2014 | How growth due to infant nutrition influences obesity and later disease risk | Review article |
| Brockway 2024 | Human Milk Macronutrients and Child Growth and Body Composition in the First Two Years: A Systematic Review | Review article |
| Callaghan 2017 | Sufficient Protein Quality of Food Aid Varies with the Physiologic Status of Recipients. | Observational study |
| Camier 2021 | Early growth according to protein content of infant formula: Results from the EDEN and ELFE birth cohorts | Cohort Study |
| Campbell 2017 | Early Life Protein Intake: Food Sources, Correlates, and Tracking across the First 5 Years of Life | Observational study |
| Chen 2021 | A Microbiota-Directed Food Intervention for Undernourished Children | Microbiota supplemented energy density food |
| Cheng 2017 | Lactoferrin and lysozyme to reduce environmental enteric dysfunction and stunting in Malawian children: study protocol for a randomized controlled trial | Author contacted, no response |
| Cheng 2019 | Supplementation With Lactoferrin and Lysozyme Ameliorates Environmental Enteric Dysfunction: A Double-Blind, Randomized, Placebo-Controlled Trial | Author contacted, no response |
| Christian 2015 | Effect of fortified complementary food supplementation on child growth in rural Bangladesh: a cluster-randomized trial | All LNS Supplement groups received same protein with only difference in Zinc |
| Collell 2016 | Higher protein intake increases cardiac function parameters in healthy children: Metabolic programming by infant nutrition-secondary analysis from a clinical trial | Interventions started after birth |
| Collins 2008 | Carbohydrate intake is the main determinant of growth in infants born <33 weeks' gestation when protein intake is adequate | Observational study |
| Damianidi 2016 | Protein intake and source during complementary feeding and growth up to 6 years of age, secondary data evaluation from the European Childhood Obesity Project | Interventions started at 2 months |
| Damianidi 2016 | Protein intakes and their nutritional sources during the first 2 years of life: secondary data evaluation from the European Childhood Obesity Project | Interventions started at 2 months |
| Das 2020 | Dietary Magnesium, Vitamin D, and Animal Protein Intake and Their Association to the Linear Growth Trajectory of Children from Birth to 24 Months of Age: Results From MAL-ED Birth Cohort Study Conducted in Dhaka, Bangladesh | Cohort Study |
| Demmelmair 2022 | Infant Feeding Choices during the First Post-Natal Months and Anthropometry at Age Seven Years: Follow-Up of a Randomized Clinical Trial | Less than 6 months |
| deVries-tenHave 2020 | Protein intake adequacy among Nigerian infants, children, adolescents and women and protein quality of commonly consumed foods. | Review article |
| Dewey 2017 | Lipid-based nutrient supplementation in the first 1000 d improves child growth in Bangladesh: a cluster-randomized effectiveness trial | LNS Supplement with interventions started in pregnancy |
| Dube 2010 | Complementary food with low (8%) or high (12%) meat content as source of dietary iron: a double-blinded randomized controlled trial | Contact Author |
| Engelmann 1998 | Meat intake and iron status in late infancy: an intervention study | Before 2000 |
| Fabiansen 2017 | Effectiveness of food supplements in increasing fat-free tissue accretion in children with moderate acute malnutrition: A randomised 2 Ã— 2 Ã— 3 factorial trial in Burkina Faso | LNS supplement (high fat in one group- no comparison) |
| Feeney 2016 | Impact of peanut consumption in the LEAP Study: Feasibility, growth, and nutrition | Focus is on peanut allergy prevention and the protein intake levels are low (6 grams/week) and constant across groups |
| Fenton 2014 | Higher versus lower protein intake in formula-fed low birth weight infants | Cochrane review for early infancy |
| FerrÃ© 2021 | Association of Protein Intake during the Second Year of Life with Weight Gain-Related Outcomes in Childhood: A Systematic Review | Review article |
| Fink 2017 | Home- and community-based growth monitoring to reduce early life growth faltering: an open-label, cluster-randomized controlled trial. | Study doesnt report on WHZ or obesity outcomes |
| Fleddermann 2016 | Growth during early infancy and anthropometry at 4 years of age: Follow-up of the BeMIM study | Less than 6 months |
| GÃ¼ntherALB 2007 | Early protein intake and later obesity risk: which protein sources at which time points throughout infancy and childhood are important for body mass index and body fat percentage at 7 y of age? | Cohort Study |
| Galasso 2019 | Effects of nutritional supplementation and home visiting on growth and development in young children in Madagascar: a cluster-randomised controlled trial | LNS with fortified MNS- No group for comparison |
| Garden 2011 | Infant and early childhood dietary predictors of overweight at age 8 years in the CAPS population. | Cohort Study |
| Ghosh 2015 | Role of Protein and Amino Acids in Infant and Young Child Nutrition: Considerations for the Development and Delivery of High Quality Complementary Food Supplements | Review article |
| GonzÃ¡lezAcero 2020 | Effect of an innovative behavioural change strategy and small-quantity lipid-based nutrient supplements on stunting and obesity in children in Baja Verapaz, Guatemala: protocol for a randomised control trial | Study Protocol (ongoing) |
| Gonzalez-Garay 2023 | Higher versus lower protein intake in formula-fed term infants | Review article |
| Gonzalez-Garay 2023 | Higher versus lower protein intake in formula-fed term infants | Review article |
| Graham 1990 | Quality-protein maize as the sole source of dietary protein and fat for rapidly growing young children | Study done in 90s, Comparison of 10 malnourished children (case study?) |
| Graham 1993 | Quality-protein maize with a high fat content as a weaning food | N=6 recovering malnourished infants fed with different complementary food |
| Grenov 2021 | The effect of milk and rapeseed protein on growth factors in 7-8Â year-old healthy children - A randomized controlled trial | Older Children |
| Gridneva 2018 | Human Milk Casein and Whey Protein and Infant Body Composition over the First 12 Months of Lactation. | Observational study |
| Gridneva 2021 | Human milk immunomodulatory proteins are related to development of infant body composition during the first year of lactation | Observational study |
| Grote 2021 | Effect of milk protein content in Toddler formula on later BMI and obesity risk: Protocol of the multicentre randomised controlled Toddler Milk Intervention (ToMI) trial | Ongoing study (Protocol) |
| Grote 2023 | THE EFFECT OF PROTEIN CONTENT OF TODDLER MILK IN THE SECOND YEAR OF LIFE ON BMI AND GROWTH: THE TODDLER MILK INTERVENTION STUDY (TOMI) | Ongoing study (Protocol) |
| Gruszfeld 2015 | Protein intake in infancy and carotid intima media thickness at 5 years--a secondary analysis from a randomized trial | Interventions started at 2 months |
| Gruszfeld 2016 | Association of early protein intake and pre-peritoneal fat at five years of age: Follow-up of a randomized clinical trial | Interventions started at 2 months |
| Gunther 2007 | Protein intake during the period of complementary feeding and early childhood and the association with body mass index and percentage body fat at 7 y of age | Cohort Study |
| Haas 2020 | The Effects of Improved Nutrition in Early Childhood on Adolescent and Early Adulthood Body Size, Composition, Maturity, and Function: Results From the First INCAP Follow-Up Study | Study done in early 70s, village cluster |
| Haschke 2016 | Postnatal High Protein Intake Can Contribute to Accelerated Weight Gain of Infants and Increased Obesity Risk | Commentary |
| Heinig 1993 | Energy and protein intakes of breast-fed and formula-fed infants during the first year of life and their association with growth velocity: the DARLING Study | Cohort Study |
| Hess 2015 | Small-Quantity Lipid-Based Nutrient Supplements, Regardless of Their Zinc Content, Increase Growth and Reduce the Prevalence of Stunting and Wasting in Young Burkinabe Children: A Cluster-Randomized Trial | All LNS Supplement groups received same protein with only difference in Zinc |
| Hilbig 2015 | Home-made and commercial complementary meals in German infants: results of the DONALD study. | Observational study |
| Hopkins 2015 | Effects on childhood body habitus of feeding large volumes of cow or formula milk compared with breastfeeding in the latter part of infancy. | Cohort Study |
| Hoppe 2004 | Protein intake at 9 mo of age is associated with body size but not with body fat in 10-y-old Danish children | Observational study |
| Hoppe 2004 | Animal protein intake, serum insulin-like growth factor I, and growth in healthy 2.5-y-old Danish children | Observational study |
| Hull 2018 | Are Protein Levels in Infant Formula a Driving Factor for Childhood Obesity Development? | Commentary |
| Humphrey 2019 | Independent and combined effects of improved water, sanitation, and hygiene, and improved complementary feeding, on child stunting and anaemia in rural Zimbabwe: a cluster-randomised trial | multidomain intervention trial that includes water quality, sanitation, handwashing (WaSH), and nutritional interventions |
| Huybregts 2019 | Impact on child acute malnutrition of integrating small-quantity lipid-based nutrient supplements into community-level screening for acute malnutrition: A cluster-randomized controlled trial in Mali | LNS with fortified MNS- No group for comparison |
| Iannotti 2014 | Linear growth increased in young children in an urban slum of Haiti: a randomized controlled trial of a lipid-based nutrient supplement | 3 groups (2 Interventions groups - LNS with fortified MNS), control group without MNS |
| Jen 2019 | Longitudinal association of dietary protein intake in infancy and adiposity throughout childhood | Cohort Study |
| Johnston 2015 | Growth and tolerance of formula with lactoferrin in infants through one year of age: double-blind, randomized, controlled trial. | Interventions started after birth |
| Joslowski 2013 | Animal protein intakes during early life and adolescence differ in their relation to the growth hormone-insulin-like-growth-factor axis in young adulthood. | Observational study |
| Kaimila 2019 | Consumption of Animal-Source Protein is Associated with Improved Height-for-Age z Scores in Rural Malawian Children Aged 12â€“36 Months. | Observational study |
| KarlslandAkeson 1998 | Growth and nutrient intake in three- to twelve-month-old infants fed human milk or formulas with varying protein concentrations | Less than 6 months |
| KarlslandAkeson 2000 | Protein intake and metabolism in formula-fed infants given Swedish or Italian weaning foods | Intervention started before 4 months |
| Khanom 2021 | Effect of RDA Protein on Renal Function in Children with Chronic Kidney Diseases. | Children with CKD |
| Kirchberg 2020 | Impact of infant protein supply and other early life factors on plasma metabolome at 5.5 and 8 years of age: a randomized trial | Interventions started at 2 months |
| Kittisakmontri 2020 | The Impact of Dietary Protein in Complementary Foods on Infant Growth and Body Composition in a Population Facing the Double Burden of Malnutrition: Protocol for a Multicenter, Prospective Cohort Study | Cohort Study |
| Kittisakmontri 2022 | Quantity and Source of Protein during Complementary Feeding and Infant Growth: Evidence from a Population Facing Double Burden of Malnutrition | Cohort Study |
| Kittisakmontri 2022 | Potential mediators of associations between protein intake from different food sources and infant growth during the complementary feeding period; Evidence from a middle-income country | Observational study |
| Koletzko 2005 | Protein intake in the first year of life: a risk factor for later obesity? The E.U. childhood obesity project | Interventions started at 2 months |
| Koletzko 2005 | Protein intake in the first year of life: A risk factor for later obesity? | Review article |
| Koletzko 2009 | Lower protein in infant formula is associated with lower weight up to age 2 y: A randomized clinical trial | Interventions started at 2 months |
| Koletzko 2009 | Can infant feeding choices modulate later obesity risk? | Interventions started at 2 months |
| Koletzko 2010 | [Infant feeding practice and later obesity risk. Indications for early metabolic programming] | Less than 6 months |
| Koletzko 2011 | Early nutrition-impact on later health | Observational study |
| Koletzko 2016 | Long-term impact on health by infant nutrition | Interventions started at 2 months |
| Koletzko 2019 | Optimized protein intakes in term infants support physiological growth and promote long-term health | Less than 6 months |
| Krebs 2011 | Meat consumption is associated with less stunting among toddlers in four diverse low-income settings. | Observational study |
| Lartey 1999 | A randomized, community-based trial of the effects of improved, centrally processed complementary foods on growth and micronutrients status of Ghanaian infants from 6 to 12 mo of age...Weanimix, a cereal-legume blend | Before 2000 |
| Lin 2008 | An energy-dense complementary food is associated with a modest increase in weight gain when compared with a fortified porridge in Malawian children aged 6-18 months. | LNS supplement (high fat in one group- no comparison) |
| Lind 2017 | Dietary protein intake and quality in early life: impact on growth and obesity | Review article |
| Liotto 2020 | Protein content of infant formula for the healthy full-term infant | Commentary |
| Luby 2018 | Effects of water quality, sanitation, handwashing, and nutritional interventions on diarrhoea and child growth in rural Bangladesh: a cluster randomised controlled trial | multidomain intervention trial that includes water quality, sanitation, handwashing (WaSH), and nutritional interventions |
| Luque 2013 | Relation of early protein intake, overweight, waist circumference and Cardiovascular Risk Factors in European children | Review article |
| Luque 2015 | Early Programming by Protein Intake: The Effect of Protein on Adiposity Development and the Growth and Functionality of Vital Organs | Review article |
| Madrigal 2021 | Dietary Intake, Nutritional Adequacy, and Food Sources of Protein and Relationships with Personal and Family Factors in Spanish Children Aged One to <10 Years: Findings of the EsNuPI Study â€ . | Observational study |
| Maleta 2015 | Provision of 10â€“40 g/d Lipid-Based Nutrient Supplements from 6 to 18 Months of Age Does Not Prevent Linear Growth Faltering in Malawi1, 2, 3 | LNS Supplement trial |
| Michaelsen 2012 | Amount and quality of dietary proteins during the first two years of life in relation to NCD risk in adulthood. | Observational study |
| Michaelsen 2013 | Effect of protein intake from 6 to 24 months on insulin-like growth factor 1 (IGF-1) levels, body composition, linear growth velocity, and linear growth acceleration: what are the implications for stunting and wasting? | Review article |
| Milani 2023 | Protein and growth during the first year of life: a systematic review and meta-analysis | Less than 6 months |
| Mostafa 2020 | Proof-of-concept study of the efficacy of a microbiota-directed complementary food formulation (MDCF) for treating moderate acute malnutrition. | No much difference in protein MDCF Vs RUSF |
| Mutumba 2024 | Effect of lipid-based nutrient supplements on micronutrient status and hemoglobin among children with stunting: secondary analysis of a randomized controlled trial in Uganda | LNS Supplement |
| NikiÃ¨ma 2014 | Treating moderate acute malnutrition in first-line health services: an effectiveness cluster-randomized trial in Burkina Faso1234 | SQ-LNS for community management of acute malnutrition |
| Nommsen 1991 | Determinants of energy, protein, lipid, and lactose concentrations in human milk during the first 12 mo of lactation: the DARLING Study | Observational study |
| Null 2018 | Effects of water quality, sanitation, handwashing, and nutritional interventions on diarrhoea and child growth in rural Kenya: a cluster-randomised controlled trial | multidomain intervention trial that includes water quality, sanitation, handwashing (WaSH), and nutritional interventions |
| Pantoja-Mendoza 2014 | Review of complementary feeding practices in Mexican children | Review article |
| Patro-Golab 2016 | Protein concentration in milk formula, growth, and later risk of obesity: A systematic review | Less than 6 months |
| Patro-Golab 2016 | Nutritional interventions or exposures in infants and children aged up to 3 years and their effects on subsequent risk of overweight, obesity and body fat: a systematic review of systematic reviews | Review article |
| Patro-Golab 2016 | The effect of different protein concentration in infant formula on growth, body composition, and later risk of obesity: A systematic review | Review article |
| Phuka 2008 | Complementary feeding with fortified spread and incidence of severe stunting in 6- to 18-month-old rural Malawians | The longer follow up of the same trial included. |
| Phuka 2009 | Postintervention growth of Malawian children who received 12-mo dietary complementation with a lipid-based nutrient supplement or maize-soy flour | LNS Supplement |
| Pimpin 2016 | Dietary protein intake is associated with body mass index and weight up to 5 y of age in a prospective cohort of twins | Cohort Study |
| Pimpin 2018 | Sources and pattern of protein intake and risk of overweight or obesity in young UK twins | Cohort Study |
| Pimpin 2019 | Effects of animal protein supplementation of mothers, preterm infants, and term infants on growth outcomes in childhood: a systematic review and meta-analysis of randomized trials. | Review article |
| Prado 2016 | Effects of maternal and child lipid-based nutrient supplements on infant development: a randomized trial in Malawi | LNS with fortified MNS- No group for comparison |
| Premji 2006 | Higher versus lower protein intake in formula-fed low birth weight infants | Less than 6 months |
| Putet 2016 | Effect of dietary protein on plasma insulin-like growth factor-1, growth, and body composition in healthy term infants: a randomised, double-blind, controlled trial (Early Protein and Obesity in Childhood (EPOCH) study). | Interventions started after birth |
| Raiha 2002 | Whey predominant, whey modified infant formula with protein/energy ratio of 1.8 g/100 kcal: Adequate and safe for term infants from birth to four months | Less than 6 months |
| Ren 2022 | The Association of Formula Protein Content and Growth in Early Infancy: A Systematic Review and Meta-Analysis | Less than 6 months |
| Rivera 2002 | Effect of supplementary feeding on the prevention of mild-to-moderate wasting in conditions of endemic malnutrition in Guatemala. | Study between 1969 and 1977 |
| Roediger 2020 | Protein quality in readyâ€toâ€use supplementary foods for moderate wasting. | SQ-LNS for community management of acute malnutrition |
| Rolland-Cachera 1995 | Influence of macronutrients on adiposity development: a follow up study of nutrition and growth from 10 months to 8 years of age | Observational study |
| Rolland-Cachera 2012 | Role of early protein intake in obesity development | Observational study |
| SandstrÃ¶m 2008 | Effects of alpha-lactalbumin-enriched formula containing different concentrations of glycomacropeptide on infant nutrition. | Less than 6 months |
| Scaglioni 2000 | Early macronutrient intake and overweight at five years of age | Observational study |
| Schlossman 2017 | A Randomized Controlled Trial of Two Ready-to-Use Supplementary Foods Demonstrates Benefit of the Higher Dairy Supplement for Reduced Wasting in Mothers, and Differential Impact in Infants and Children Associated With Maternal Supplement Response | Focused on acute malnutrition |
| Sehgal 2020 | A Comparative Study of infant and young child feeding practices (IYCF) and nutritional status under two years of age. | Comparison of feeding practices in complementary feeding |
| Shivakumar 2019 | Protein-quality evaluation of complementary foods in Indian children | Experimental study to assess food quality |
| Smithâ€Brown 2018 | Growth and protein-rich food intake in infancy is associated with fat-free mass index at 2-3 years of age. | Cohort Study |
| Smuts 2019 | Effect of small-quantity lipid-based nutrient supplements on growth, psychomotor development, iron status, and morbidity among 6- to 12-mo-old infants in South Africa: a randomized controlled trial | LNS with fortified MNS- No group for comparison |
| Sobik 2021 | Early infant feeding effect on growth and body composition during the first 6 years and neurodevelopment at age 72 months. | Observational study |
| Socha 2011 | Milk protein intake, the metabolic-endocrine response, and growth in infancy: data from a randomized clinical trial | Interventions started after birth |
| Socha 2016 | Endocrine and Metabolic Biomarkers Predicting Early Childhood Obesity Risk | Less than 6 months |
| Soofi 2019 | Effect of Lipid-based Nutrient Supplement - Medium Quantity on Reduction of Stunting in Children 6â€“23 Months of Age in Sindh, Pakistan: A Cluster Randomized Controlled Trial (OR25-06-19) | LNS with fortified MNS- No group for comparison |
| Spalinger 2017 | Growth of Infants Fed Formula with Evolving Nutrition Composition: A Single-Arm Non-Inferiority Study. | Interventions started after birth |
| Stobaugh 2016 | Including whey protein and whey permeate in ready-to-use supplementary food improves recovery rates in children with moderate acute malnutrition: a randomized, double-blind clinical trial1, 2, 3 | Focused on acute malnutrition |
| Svahn 1999 | Macronutrient and energy intakes in young children fed milk products containing different quantities and qualities of fat and protein | Before 2000 |
| Tang 2016 | Dietary intakes of formula-fed infants consuming a meat-or dairy-based complementary diet: A semi-controlled feeding trial | Tang et. al study already included |
| Tang 2018 | Protein intake during the first two years of life and its association with growth and risk of overweight | Review article |
| Tang 2021 | Effects of Complementary Feeding With Different Protein-Rich Foods on Infant Growth and Gut Health: Study Protocol | Ongoing study (Protocol) |
| Thakwalakwa 2015 | Impact of lipid-based nutrient supplements and corn-soy blend on energy and nutrient intake among moderately underweight 8-18-month-old children participating in a clinical trial | Energy source (lipids for LNS, carbohydrates for CSB), and different micronutrient content |
| Thorisdottir 2014 | Animal protein intake at 12 months is associated with growth factors at the age of six. | Cohort Study |
| Tincu 2016 | How early nutrition modify gain weight in the first 2 years of life | Observational study |
| Tincu 2019 | Influence of protein intake in the first year of life on body size and igf-i levels | Commentary |
| Tincu 2020 | Influence of protein intake during complementary feeding on body size and IGF-I levels in twelve-month-old infants | Commentary |
| Totzauer 2018 | Effect of Lower Versus Higher Protein Content in Infant Formula Through the First Year on Body Composition from 1 to 6 Years: Follow-Up of a Randomized Clinical Trial. | Interventions started at 2 months |
| Uauy 2015 | Role of Protein and Amino Acids in Infant and Young Child Nutrition: Protein and Amino Acid Needs and Relationship with Child Growth | Review article |
| Udoh 2022 | Comparison of standardised milkâ€based, standardised nonâ€milk based and hospitalâ€based formulations on the anthropometric indices of underâ€fives with moderate acute malnutrition: A randomised clinical trial. | Focused on acute malnutrition |
| vanÂ Vliet 2022 | The Baby's First Bites RCT: Evaluating a Vegetable-Exposure and a Sensitive-Feeding Intervention in Terms of Child Health Outcomes and Maternal Feeding Behavior During Toddlerhood. | Vegetable consumption |
| vanderVeek 2019 | Baby's first bites: a randomized controlled trial to assess the effects of vegetable-exposure and sensitive feeding on vegetable acceptance, eating behavior and weight gain in infants and toddlers. | Vegetable consumption |
| Verduci 2018 | Feeding cows' milk or follow-on formula for young children in the second year of life: Observational data from the Childhood Obesity Project (CHOP) | Interventions started at 2 months |
| Verduci 2022 | Different protein intake in the first year and its effects on adiposity rebound and obesity throughout childhood: 11 years follow-up of a randomized controlled trial | Interventions started at 2 months |
| Voortman 2016 | Protein intake in early childhood and cardiometabolic health at school age: the Generation R Study. | Cohort Study |
| Voortman 2016 | Protein intake in early childhood and body composition at the age of 6 years: The Generation R Study | Cohort Study |
| Weber 2014 | Lower protein content in infant formula reduces BMI and obesity risk at school age: Follow-up of a randomized trial | Interventions started at 2 months |
| Weber 2016 | Effect of early protein supply on body fat deposition during infancy and childhood: A randomized trial | Interventions started at 2 months |
| Weijs 2011 | High beverage sugar as well as high animal protein intake at infancy may increase overweight risk at 8 years: a prospective longitudinal pilot study. | Cohort Study |
| Wright 2016 | Breastfeeding and Protein Intake Influence Body Mass Index from 2 Months to 22 Years in the Cebu Longitudinal Health and Nutrition Survey | Observational study |
| Yang 2013 | Nutrition in pregnancy and early childhood and associations with obesity in developing countries | Observational study |
| Zheng 2021 | Protein Intake During Infancy and Subsequent Body Mass Index in Early Childhood: Results from the Melbourne InFANT Program | Cohort Study |

**Section 2:** **Narrative Results**

**High Vs Low Protein Supplementation**

Agapova et al. compared high-protein diets using common bean flour and cowpea flour with a control group receiving corn-soy blend in Malawian children aged 6–23 months. No significant differences in linear growth outcomes or WLZ were found between the groups(1). Ghosh et al. compared KOKO Plus, a lysine-enriched high-protein supplement, with a micronutrient powder in Bangladeshi children aged 6–24 months. KP supplementation led to significant improvements in linear growth, but no significant differences in WHZ were observed between the groups(2).

Iannotti et al. in rural Ecuador children aged 6–9 months with moderate stunting provided egg to the intervention group which showed significant improvements in linear growth and WLZ compared to the standard of care group (3). Johansson et al. compared a protein-reduced complementary diet to conventional dietary recommendations in Swedish infants from 6 months of age. The reduced protein group had significantly lower fat mass and fat mass index at 12 months, without compromising fat-free mass or linear growth. IGF-1 levels were also significantly lower in the reduced protein group in both 12 and 18 months (4) (means and SDs not reported). In contrast, plasma insulin concentrations did not differ significantly between groups at either time point. Konyole et al. evaluated locally produced complementary foods with varying protein levels, including high-protein formulations containing animal-source foods (ASF) and lower-protein plant-based formulations, in Kenyan infants aged 6–15 months. No significant differences in FFM gain, length gain, or WLZ were observed between the groups (5). Larnkjær et al. compared whole milk (22% protein energy) with infant formula (10% protein energy) on growth and IGF-I concentrations in infants aged 9 to 12 months. No significant differences in weight, length, or weight gain were observed between the groups, though PE% intake was positively correlated with IGF-I levels (6) compared to the formula group, and protein energy percentage intake was positively correlated with IGF-1 levels. Insulin was not measured in this study

Mangani et al. assessed complementary feeding interventions using supplements with either milk or soy protein, and Corn Soy Blend (CSB) compared to a control group in rural Malawi. No significant differences in stunting reduction or linear growth were observed between the groups(7).

Ochoa et al. compared a Fish Protein Isolate diet to a standard animal protein diet in Peruvian children aged 6-36 months. No significant differences in HAZ and WHZ were found between the groups(8). Stephenson et al. assessed the impact of cowpea and common bean-based complementary foods compared to a control group in Malawian children. No significant differences in WHZ were observed between the groups(9).

Taneja et al. compared high protein milk-cereal supplementation with no supplementation (one comparison) in infants between 6 and 12 months and found no significant difference in fat mass or fat-free mass, but an improvement in LAZ at 12 months (10). Wall et al. compared Growing Up Milk Lite with reduced protein content to standard whole cow milk in healthy 1-year-olds. The GUMLi group had significantly lower body fat and fat mass index, with no significant differences in fat-free mass or linear growth, suggesting healthier body composition with reduced protein intake (11).

The Ghosh et al, conducted in Ghana, compared a macro- and micronutrient-fortified supplement (KOKO Plus) with a micronutrient powder and found no significant differences in WLZ between groups at endline. This study was not included in the quantitative meta-analysis as the absolute difference in protein intake between intervention arms was less than 5 grams per day, which did not meet the prespecified inclusion threshold for high versus low protein comparisons.

**Animal Vs Plant protein source**

Tang and Krebs et al. examined rural Chinese breastfed infants receiving pureed meats compared to cereals. The meat group showed significant improvements in LAZ but no significant differences in WLZ, indicating no increase in adiposity (12). Similarly, Krebs et al. observed a trend toward improved LAZ in infants receiving lyophilized beef compared to fortified rice-soy cereal, with no significant differences in WLZ between the groups (13).

Mbabazi et al. compared milk protein and whey permeate with soy protein and maltodextrin, finding a small but significant improvement in FFMI for the milk protein group. However, there were no significant differences in FMI or WLZ between groups, suggesting no impact on adiposity (14). Tang et al. (2014, Rural China) and Tang et al. (2014, USA) compared meat-based complementary foods to cereal-based diets in breastfed infants. Both studies found that the meat group showed significant improvements in WAZ and LAZ. No significant differences in WLZ or adiposity measures, suggesting that animal protein promotes linear growth without contributing to excess fat deposition compared to plant-based sources(12, 15).

**Meat vs. Dairy Comparisons**

In infants aged 5–12 months, diets based on dairy protein resulted in higher WLZ, reflecting greater relative weight gain, whereas meat-based diets showed growth patterns suggestive of improved linear development (Tang et al.). No significant differences were observed in adiposity-related markers, including IGF-1 or BMI, during the intervention(16). At 24 months, the difference in WLZ between the groups was no longer significant, while the meat-based group continued to display growth patterns favoring linear growth. No differences in IGF-1 levels or other adiposity measures were identified at the follow-up, suggesting no long-term impact of protein source on adiposity(17).

Skau et al. compared fortified complementary food products in Cambodia, finding no significant differences in WLZ between fish-based and milk/soy-based foods. Both groups showed improvements in linear growth relative to their own baseline measures, with similar fat-free mass and fat mass between groups(18). Borg et al. evaluated isocaloric and isoproteic ready-to-use supplementary foods in Cambodia, finding no significant differences in WLZ or LAZ outcomes between fish-based and milk/soy-based foods, suggesting that protein source did not differentially impact growth(19).

Long et al. investigated meat and milk porridges in Kenyan toddlers and reported that milk porridge promoted greater linear growth compared to meat porridge, though no differences in overall energy intake were observed between groups(20).

Table S3: Summary for High vs. Low Protein Supplementation studies – Quantitative Synthesis

| **study_id** | **country** | **isocaloric** | **qu_high** | **qu_low** | **Type** | **Baseline diet** | **Difference (g)** |
| --- | --- | --- | --- | --- | --- | --- | --- |
| **Supplement Based** | | | | | | | |
| Agapova 2018 | Low to Middle | No | Common beans 24–27% of energy from protein (13g in supplement) | CSB around 14% energy from protein (+6.5g in supplement) | Additional Supplement (Legume-based supplement vs. cereal blend) | Similar | 6.5 |
| Mangani 2015 | Low to Middle | No | Corn Soy Blend + (+10.4 g in supplement) | No Suppl | Additional Supplement (CSB+ : CSB with added dairy and micronutrients) | Not mentioned | 10.4 |
| Taneja 2022 | Low to Middle | No | Milk Cereal protein Overall 17.6 g / day) | No supplementation (overall 11.5 g / day) | Additional Supplement (milk Cereal Mix) | Similar | 6.1 |
| **Dietary Modification** | | | | | | | |
| Johansson 2023 | High | Yes | Conventional (swedish) Overall 28.4 g / day | Nordic (low protein): Overall 20 g/ day | Diet Modification (Nordic focusing more on plant-based foods and whole grains) | Not similar | 8.4 |
| Long 2012 | Low to Middle | Yes | Meat porridge (13g protein); overall no information | Milk porridge (5.9g protein) overall no information | Diet Modification | Not similar | 7.1 |
| Wall 2019 | High | Yes | Whole Cow Milk (3.1 g/100ml) & overall 51 g / day | Growing Up Milk Lite (GUMLi) with reduced protein content (1.7 g/100 mL) (red. Protein) & Overall 46 g / day | Diet Modification | Not similar | 5 |

## Figure S1a: Risk of Bias (RoB) for Individually Randomized Trials

| Study ID | **D1** | **D2** | **D3** | **D4** | **D5** | **Overall** |
| --- | --- | --- | --- | --- | --- | --- |
| Johansson 2023 |  |  |  |  |  |  |
| Taneja 2019 |  |  |  |  |  |  |
| Wall 2019 |  |  |  |  |  |  |
| Tang 2018 |  |  |  |  |  |  |
| Skau 2015 |  |  |  |  |  |  |
| Long 2012 |  |  |  |  |  |  |
| Mangani 2015 |  |  |  |  |  |  |
| Mbabazi 2023 |  |  |  |  |  |  |

## Figure S1b: Risk of Bias (RoB) for Cluster-Randomized Trials

| **Unique ID** | **D1a** | **D1b** | **D2** | **D3** | **D4** | **D5** | **Overall** |
| --- | --- | --- | --- | --- | --- | --- | --- |
| Agapova 2018 |  |  |  |  |  |  |  |
| Borg 2020 |  |  |  |  |  |  |  |
| Tang 2014 |  |  |  |  |  |  |  |
| Krebs 2012 |  |  |  |  |  |  |  |

| \|  \| \| --- \| | Low risk |
| --- | --- | --- |
| \|  \| \| --- \| | Some concerns |
| \|  \| \| --- \| | High risk |

| D1 | Randomization process | D1a | Randomization process |
| --- | --- | --- | --- |
| D2 | Deviations from the intended interventions | D1b | Timing of identification or recruitment of participants |
| D3 | Missing outcome data | D2 | Deviations from the intended interventions |
| D4 | Measurement of the outcome | D3 | Missing outcome data |
| D5 | Selection of the reported result | D4 | Measurement of the outcome |
|  |  | D5 | Selection of the reported result |

Figure S1c. Weighted risk of bias summary: individually randomized trials.


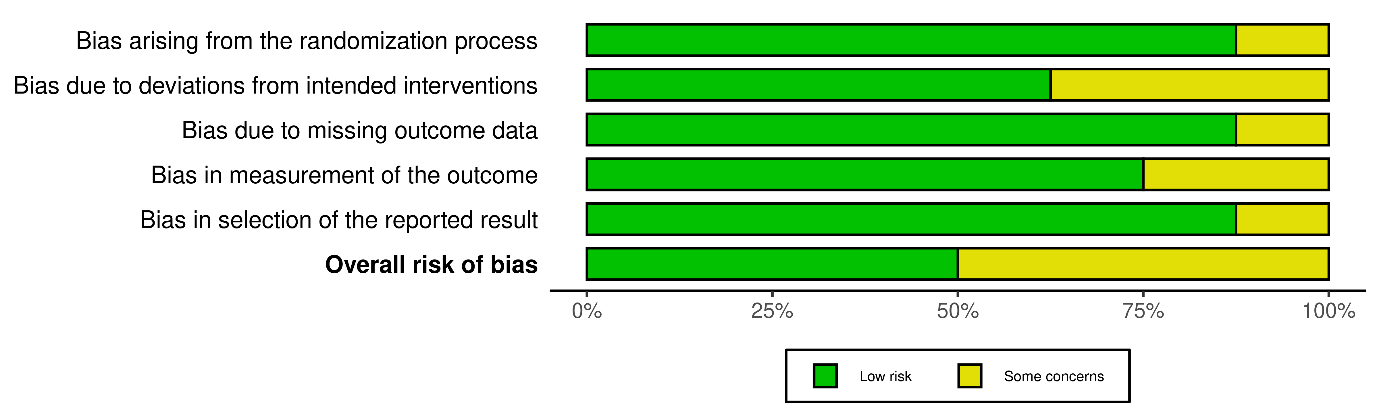


Figure S1d. Weighted risk of bias summary: cluster-randomized trials.


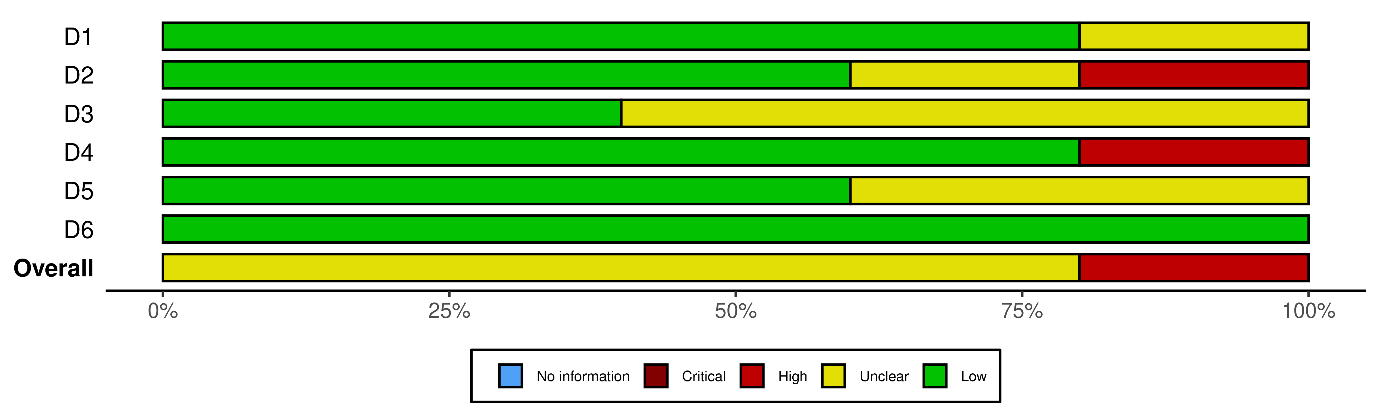


Figure S2: Sensitivity analysis: Meat compared with dairy-based protein and weight-for-length z-score (WLZ) after excluding the high risk of bias study.


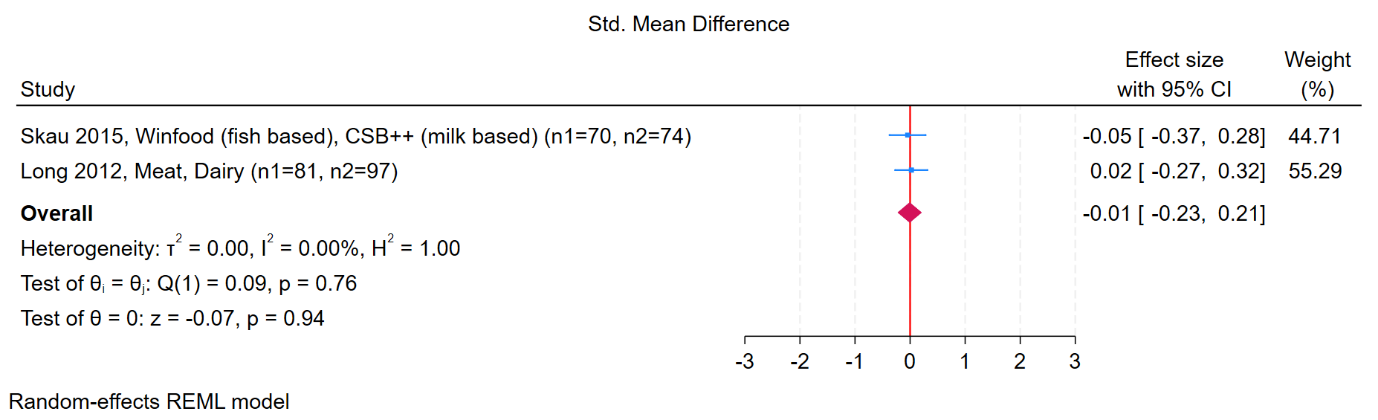


Forest plot showing the effect of meat compared with dairy-based protein intake during complementary feeding on weight-for-length z-score (WLZ), after excluding the study rated at high risk of bias. The plot presents standardized mean differences (SMDs) with 95% confidence intervals for the remaining studies. No significant differences were observed in WLZ after removal of the high risk of bias study.

Table S4: GRADE Summary for High Protein Supplementation compared to Low Protein Supplementation

| **Certainty assessment** | | | | | | | **№ of patients** | | **Effect** | | **Certainty** | **Importance** |
| --- | --- | --- | --- | --- | --- | --- | --- | --- | --- | --- | --- | --- |
| **№ of studies** | **Study design** | **Risk of bias** | **Inconsistency** | **Indirectness** | **Imprecision** | **Other considerations** | **High Protein Supplementation** | **No Supplementation** | **Relative (95% CI)** | **Absolute (95% CI)** |  |  |
| **Weight for Length Z score (follow-up: mean 12)** | | | | | | | | | | | | |
| 5 | randomized trials | serious^a^ | serious^b^ | serious^c^ | serious^d^ | none | 1177 | 1183 | - | SMD **0.02 SD lower**  (-0.10 lower to 0.06 higher) | ⨁◯◯◯ Very low^a,b,c,d^ | IMPORTANT |
| **Fat Mass** | | | | | | | | | | | | |
| 2 | randomized trials | serious | serious^g^ | serious^h^ | serious^i^ | none | 127 | 109 | - | SMD **0.17 SD higher** (-0.41 lower to 0.74 higher) | ⨁◯◯◯ Very low^g,h,i^ | IMPORTANT |
| **Fat Free Mass (follow-up: mean 12)** | | | | | | | | | | | | |
| 2 | randomized trials | serious | serious | serious | serious | none | 127 | 109 | - | SMD **0.05 SD lower** (0.31 lower to 0.21 higher) | ⨁◯◯◯ Very low |  |

**CI:** confidence interval; **SMD:** standardized mean difference

#### Explanations

a. Downgraded one level due to some concerns in RoB of most of the studies

b. Downgrade by 1 level for substantial heterogeneity

c. Downgrade by 1 level for differences in population, intervention, and comparator across studies.

d. Downgrade by 1 level due to wide confidence intervals and lack of statistical significance

e. Downgraded two levels for 2 studies with some concerns and one with high risk of bias

f. Downgraded due to wide CIs that fail to exclude meaningful effects in either direction.

g. Downgrade the inconsistency by one level due to moderate heterogeneity and opposing effects

h. Downgraded due to variation in populations, interventions, and potentially outcome measurement methods.

i. Downgraded due to wide confidence intervals, and crossing the null effect.

Table S5: GRADE Summary for Meat vs. Dairy Protein Supplementation

| **Certainty assessment** | | | | | | | **№ of patients** | | **Effect** | | **Certainty** | **Importance** |
| --- | --- | --- | --- | --- | --- | --- | --- | --- | --- | --- | --- | --- |
| **№ of studies** | **Study design** | **Risk of bias** | **Inconsistency** | **Indirectness** | **Imprecision** | **Other considerations** | **Meat** | **Dairy** | **Relative (95% CI)** | **Absolute (95% CI)** |  |  |
| **Weight for Length Z score (follow-up: mean 24 months)** | | | | | | | | | | | | |
| 3 | randomized trials | serious^a^ | serious^b^ | serious^c^ | serious^d^ | none | 308 | 327 | - | SMD **0.04 SD lower**  (0.22 lower to 0.15 higher) | ⨁◯◯◯ Very low^a,b,c,d^ | IMPORTANT |

**CI:** confidence interval; **SMD:** standardized mean difference

#### Explanations

a. The overall risk of bias was downgraded due to the presence of one study with a high risk of bias, one study with some concerns

b. Inconsistency was downgraded by one level due to high heterogeneity and significant subgroup differences

c. Indirectness was downgraded by one level due to variations in intervention composition and delivery methods across studies

d. Imprecision was downgraded by one level due to wide confidence intervals that span the threshold for no effect and include both clinically significant negative and positive effects

Table S6: GRADE Summary for Animal vs. Plant Protein Supplementation

| **Certainty assessment** | | | | | | | **№ of patients** | | **Effect** | | **Certainty** | **Importance** |
| --- | --- | --- | --- | --- | --- | --- | --- | --- | --- | --- | --- | --- |
| **№ of studies** | **Study design** | **Risk of bias** | **Inconsistency** | **Indirectness** | **Imprecision** | **Other considerations** | **Animal Protein** | **Plant protein** | **Relative (95% CI)** | **Absolute (95% CI)** |  |  |
| **Weight for Length Z score (follow-up: mean 12)** | | | | | | | | | | | | |
| 4 | randomized trials | serious^a^ | not serious^b^ | not serious | serious^c^ | none | 1497 | 1897 | - | SMD **0.01 SD higher** (0.06 lower to 0.09 higher) | ⨁⨁◯◯ Low^a,b,c^ | IMPORTANT |

**CI:** confidence interval; **SMD:** standardized mean difference

#### Explanations

a. RoB has downgraded by one, due to some concerns in 2 of the studies

b. Studies showed consistent findings with low heterogeneity and closely aligned effect sizes, indicating no substantial inconsistency

c. While the CI is relatively narrow, it includes the null value and does not provide strong evidence for or against a true effect. hence downgrading by one level for imprecision

**REFERENCES**

1. Agapova SE, Stephenson KB, Divala O, Kaimila Y, Maleta KM, Thakwalakwa C, Ordiz MI, Trehan I, Manary MJ. Additional Common Bean in the Diet of Malawian Children Does Not Affect Linear Growth, but Reduces Intestinal Permeability. J Nutr. 2018;148(2):267-74. doi: 10.1093/jn/nxx013.

2. Ghosh SA, Strutt NR, Otoo GE, Suri DJ, Ankrah J, Johnson T, et al. A macro- and micronutrient-fortified complementary food supplement reduced acute infection, improved haemoglobin and showed a dose-response effect in improving linear growth: a 12-month cluster randomised trial. J Nutr Sci. 2019;8:e22. doi: 10.1017/jns.2019.18.

3. Iannotti LL, Lutter CK, Stewart CP, Gallegos Riofrío CA, Malo C, Reinhart G, et al. Eggs in Early Complementary Feeding and Child Growth: A Randomized Controlled Trial. Pediatrics. 2017;140(1). doi: 10.1542/peds.2016-3459.

4. Johansson U, Öhlund I, Lindberg L, Hernell O, Lönnerdal B, Venables M, Lind T. A randomized, controlled trial of a Nordic, protein-reduced complementary diet in infants: effects on body composition, growth, biomarkers, and dietary intake at 12 and 18 months. Am J Clin Nutr. 2023;117(6):1219-31. doi: 10.1016/j.ajcnut.2023.03.020.

5. Konyole SO, Omollo SA, Kinyuru JN, Skau JKH, Owuor BO, Estambale BB, et al. Effect of locally produced complementary foods on fat-free mass, linear growth, and iron status among Kenyan infants: A randomized controlled trial. Maternal & child nutrition. 2019;15(4):e12836. doi: 10.1111/mcn.12836.

6. Larnkjaer A, Hoppe C, Mølgaard C, Michaelsen KF. The effects of whole milk and infant formula on growth and IGF-I in late infancy. Eur J Clin Nutr. 2009;63(8):956-63. doi: 10.1038/ejcn.2008.80.

7. Mangani C, Maleta K, Phuka J, Cheung YB, Thakwalakwa C, Dewey K, Manary M, Puumalainen T, Ashorn P. Effect of complementary feeding with lipid-based nutrient supplements and corn-soy blend on the incidence of stunting and linear growth among 6- to 18-month-old infants and children in rural Malawi. Maternal & child nutrition. 2015;11 Suppl 4(Suppl 4):132-43. doi: 10.1111/mcn.12068.

8. Ochoa TJ, Baiocchi N, Valdiviezo G, Bullon V, Campos M, Llanos-Cuentas A. Evaluation of the efficacy, safety and acceptability of a fish protein isolate in the nutrition of children under 36 months of age. Public Health Nutr. 2017;20(15):2819-26. doi: 10.1017/s136898001700163x.

9. Stephenson KB, Agapova SE, Divala OH, Kaimila Y, Maleta KM, Thakwalakwa C, Ordiz MI, Trehan I, Manary MJ. Complementary feeding with cowpea reduces growth faltering in rural Malawian infants: a blind, randomized controlled clinical trial. The American journal of clinical nutrition. 2017;106 6:1500-7.

10. Manapurath R, Chowdhury R, Upadhyay RP, Kurpad AV, Bose B, Devi S, Dwarkanath P, Bhandari N, Taneja S, Strand TA. Impact of high- and moderate-protein supplementation on early-life obesity and body composition: a randomized controlled trial in India. Am J Clin Nutr. 2025;121(6):1380-6. doi: 10.1016/j.ajcnut.2025.04.007.

11. Wall CR, Hill RJ, Lovell AL, Matsuyama M, Milne T, Grant CC, Jiang Y, Chen RX, Wouldes TA, Davies PSW. A multicenter, double-blind, randomized, placebo-controlled trial to evaluate the effect of consuming Growing Up Milk "Lite" on body composition in children aged 12-23 mo. Am J Clin Nutr. 2019;109(3):576-85. doi: 10.1093/ajcn/nqy302.

12. Tang M, Krebs NF. High protein intake from meat as complementary food increases growth but not adiposity in breastfed infants: a randomized trial. Am J Clin Nutr. 2014;100(5):1322-8. doi: 10.3945/ajcn.114.088807.

13. Krebs NF, Mazariegos M, Chomba E, Sami N, Pasha O, Tshefu A, et al. Randomized controlled trial of meat compared with multimicronutrient-fortified cereal in infants and toddlers with high stunting rates in diverse settings. Am J Clin Nutr. 2012;96(4):840-7. doi: 10.3945/ajcn.112.041962.

14. Mbabazi J, Pesu H, Mutumba R, Filteau S, Lewis JI, Wells JC, et al. Effect of milk protein and whey permeate in large quantity lipid-based nutrient supplement on linear growth and body composition among stunted children: A randomized 2 × 2 factorial trial in Uganda. PLoS Med. 2023;20(5):e1004227. doi: 10.1371/journal.pmed.1004227.

15. Tang M, Sheng XY, Krebs NF, Hambidge KM. Meat as complementary food for older breastfed infants and toddlers: a randomized, controlled trial in rural China. Food Nutr Bull. 2014;35(4 Suppl):S188-92. doi: 10.1177/15648265140354s304.

16. Tang M, Hendricks AE, Krebs NF. A meat- or dairy-based complementary diet leads to distinct growth patterns in formula-fed infants: a randomized controlled trial. Am J Clin Nutr. 2018;107(5):734-42. doi: 10.1093/ajcn/nqy038.

17. Tang M, Andersen V, Hendricks AE, Krebs NF. Different Growth Patterns Persist at 24 Months of Age in Formula-Fed Infants Randomized to Consume a Meat- or Dairy-Based Complementary Diet from 5 to 12 Months of Age. J Pediatr. 2019;206:78-82. doi: 10.1016/j.jpeds.2018.10.020.

18. Skau JK, Touch B, Chhoun C, Chea M, Unni US, Makurat J, et al. Effects of animal source food and micronutrient fortification in complementary food products on body composition, iron status, and linear growth: a randomized trial in Cambodia. Am J Clin Nutr. 2015;101(4):742-51. doi: 10.3945/ajcn.114.084889.

19. Borg B, Mihrshahi S, Griffin M, Sok D, Chhoun C, Laillou A, Berger J, Wieringa FT. Randomised controlled trial to test the effectiveness of a locally-produced ready-to-use supplementary food (RUSF) in preventing growth faltering and improving micronutrient status for children under two years in Cambodia: a study protocol. Nutr J. 2018;17(1):39. doi: 10.1186/s12937-018-0346-x.

20. Long JK, Murphy SP, Weiss RE, Nyerere S, Bwibo NO, Neumann CG. Meat and milk intakes and toddler growth: a comparison feeding intervention of animal-source foods in rural Kenya. Public Health Nutr. 2012;15(6):1100-7. doi: 10.1017/s1368980011002746.
